# Supplementary material for: Self-assembled photonic cavities with atomic-scale confinement
Source: Nature. 2023 Dec 6;624(7990):57–63. doi: 10.1038/s41586-023-06736-8 (PMC10700130; doi:10.1038/s41586-023-06736-8)
Supplement: Supplementary file 1 — This file contains Supplementary Sections S1–S5, including Supplementary Figs. 1–34 and Supplementary Tables 1–3. [file 41586_2023_6736_MOESM1_ESM.pdf]

nature portfolio

https://doi.org/10.1038/s41586-023-06736-8

Supplementary information

Self-assembled photonic cavities with atomic-scale confinement

In the format provided by the authors and unedited

Supplementary information for
Self-assembled photonic cavities with atomic-scale confinement

Ali Nawaz Babar,[1,2,*] Thor August Schimmell Weis,[1] Konstantinos Tsoukalas,[1] Shima Kadkhodazadeh,[2,3] Guillermo Arregui,[1] Babak Vosoughi Lahijani,[1,2] and Søren Stobbe[1,2,†]

[1]*Department of Electrical and Photonics Engineering,*
DTU Electro, Technical University of Denmark,
Building 343, DK-2800 Kgs. Lyngby, Denmark

[2]*NanoPhoton - Center for Nanophotonics, Technical University of Denmark,*
Ørsteds Plads 345A, DK-2800 Kgs. Lyngby, Denmark.

[3]*DTU Nanolab, Technical University of Denmark,*
Building 347, DK-2800 Kgs. Lyngby, Denmark

S1. Circumventing the resolution limit of top-down nanofabrication **2**

S1.1. Definition of resolution 2

S1.2. The resolution limit in self-assembled devices 3

S2. Investigation of pull-in instabilities due to surface forces **6**

S2.1. The effect of built-in stress release 6

S2.2. Numerical simulations for extracting the spring constant 9

S2.3. Mapping the threshold for self-assembly by surface forces 9

S2.4. The critical gap of Casimir-Lifshitz theory as the threshold for self-assembly by surface forces 14

S3. Design of nanobeam photonic-crystal cavities with bowtie unit cells **17**

S3.1. Band structures and cavity design 17

S3.2. Evaluation of the effective mode volume 19

S3.3. Numerical calculations and mesh convergence of nanobeam bowtie cavities. 21

S3.4. The role of the native oxide 25

S3.5. The role of disorder 27

S4. Self-assembly of air-bowtie cavities **29**
S4.1. Silicon etch and sidewall verticality 29
S4.2. Deterministic fabrication and size control of air bowties 33
S4.3. Scanning electron microscope characterization of offset-to-width correspondence 36
S4.4. Scanning transmission electron microscope characterization of bowtie widths 39
S4.5. Self-assembled nanobeam bowtie cavities 42

S5. Optical spectroscopy of air-bowtie nanocavities **42**
S5.1. Far-field resonant scattering measurements 42
S5.2. In-line transmission measurements of self-assembled nanobeam cavities 44

References **54**

S1. CIRCUMVENTING THE RESOLUTION LIMIT OF TOP-DOWN NANOFABRICATION

S1.1. Definition of resolution

In the semiconductor industry, the term “resolution” is defined as the smallest critical dimension, i.e., the width of the smallest line that can be reliably fabricated. The resolution must not be confused with the precision with which the edge of the lines can be reproducibly placed, as the latter is related to the standard deviation of the resolution rather than the resolution itself. The resolution must also not be confused with the number used to name the so-called technology nodes. For example, the current “3 nm node” [1] uses a fabrication process with a 12 nm resolution. The current industry roadmap [2] forecasts no lateral lithography features (minimum half-pitch or physical gate length) below 8 nm for the next 15 years. Strictly speaking, the numbers in the roadmap are half-pitches of very large arrays of equally sized positive (ridges) and negative (gaps) lines, i.e., the half-pitch is the same as the width of a single line. Here, we refer to single gaps rather than arrays of gaps and lines, but comparing the achieved resolution of our self-assembly process to that of conventional top-down nanofabrication is fair in this case because it is much harder to fabricate gaps than

ridges (see Section 4.1).

S1.2. The resolution limit in self-assembled devices

Figure S1 shows a schematic illustration of how our self-assembly method circumvents the resolution limitation of conventional lithography and etching. This relies on the way in which mask dimensions, process variations, and surface roughness affect the resolution of the self-assembly method. Figure S1a illustrates a single trench of width s in the lithography mask, where the width of the line is at least as large as the resolution of the top-down process, $s_{\min}$. The edge of the line in the fabricated device will be exactly at the mask edge except for two undesirable effects: a random local variation with zero mean due to surface roughness, δs, and a global shift due to global drifts in parameters for the particular fabrication batch, Δe, due to, e.g., day-to-day variations in resist thickness, developer temperature, gas flows in the dry etcher, etc. Figure S1b shows the effect of local disorder and global shifts when fabricating a bowtie structure, which is afterwards self-assembled into the bowtie shown in Fig. S1c. It is important to stress that the mask shown in Fig. S1b differs from the mask in our experiment (see Fig. 2 in the main text and Section S4.2) on an important point: It obeys the design rules. In our experiment, our mask pattern employed sharp tips to get the smallest possible solid radius of curvature in the final devices. In a foundry, the shape would be designed within the limits of the design rules. This means that, in our particular realization of the self-assembly protocol, there are additional effects of mask erosion and proximity effects at the tips of the bowties, but these effects are not fundamental to our concepts. When operating within the borders of the design rules, the global shift, Δe, will be conformal. Regardless of whether the devices are made in a university cleanroom or produced in a foundry, the important point of the self-assembly protocol is that the final gap, g (the width of the bowtie center in Fig. S1c), does not depend on Δe and neither does it depend on the resolution of the top-down process, $s_{\min}$. The gap depends on the distance between the edges of the gap bounding the bowtie shape and the bowtie tips. These distances can be fine-tuned by, e.g., locally varying the electron-beam dose, which can be done nearly continuously. As a consequence, g is ultimately limited by the roughness δs.

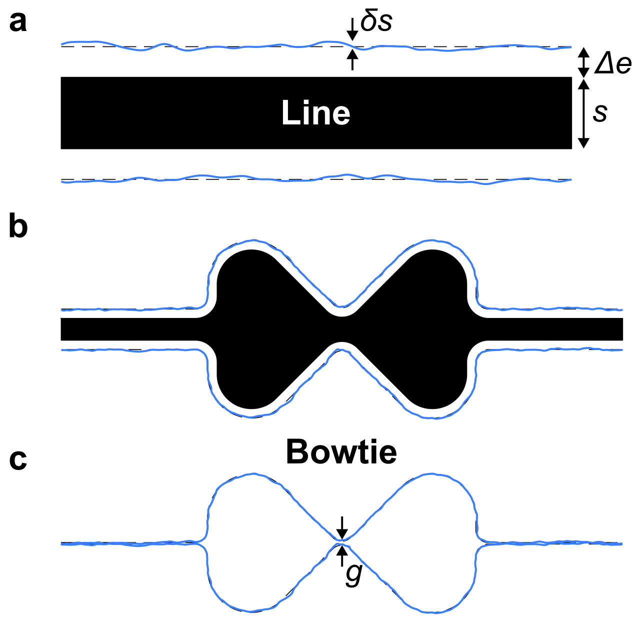

Supplementary Figure S1. Schematic illustration of the deviations between lithography mask and resulting devices and why the gaps in our self-assembled cavities are independent of the resolution. a, A gap is as a line (black) between two parallel silicon edges. The lithography mask (solid lines) is transferred into the silicon along with two kinds of unavoidable errors: Structural disorder of mean zero (random lines) varying around a global shift (dashed lines). The resolution (not shown) is defined as the smallest linewidth that can be reliably fabricated, and the combined effect of the global shift and the structural disorder determines the total error of devices made exactly with the smallest possible critical dimension. b, When fabricating bowtie cavities by our method in a foundry setting, the mask would be designed within the boundaries of the fabrication constraints, i.e., with roundings reflecting the smallest possible solid and void radii of curvature. This means that the global shift moves the silicon edge in a conformal manner while the structural disorder is a random function. c, The same structure after self-assembly results in a gap determined by the offset between the planar sides and the tip, but this cannot be made smaller than the disorder, which, for a given fabrication process, limits the smallest possible devices that our method allows fabricating. In a state-of-the-art foundry setting, both the global shift and the amplitude of the disorder are below the size of a single silicon atom.

The self-assembly method we propose uses technology already available in most smaller foundries and well-equipped university cleanrooms. The self-assembly method obviously requires the devices to be underetched, but for nanophotonic and nanomechanics, this un-

deretching is only an advantage (e.g., improved optical confinement in nanocavities and silicon photonics) or even a requirement (nanoelectromechanical systems, optomechanics, gallium-arsenide quantum photonics for which there is no low-index substrate, etc.). Since the resolution of our process is limited by roughness, the question is what the amplitude of the roughness is in a scalable production setting, i.e., in a semiconductor foundry. To address that question, we refer to Panuski *et al.* [3], who present a study of photonic nanocavities fabricated in a foundry, notably using silicon membranes of the same thickness as ours. More specifically, Fig. 4d in Panuski *et al.* presents measured distributions of the resonant wavelengths and quality factors on large ensembles of several types of silicon photonic-crystal cavities. These ensemble measurements contain information about the combined effects of the global shift of the silicon edges, structural disorder, and all other deviations from the mathematically perfect structure. We consider the worst-case scenario they report, namely that of L4/3 cavities [4], for which the standard deviation in the resonance wavelength distribution is measured to be 1.1 nm. It is easy to estimate how this translates into structural deformations by considering a simple example: If the silicon edge grows by 1 nm, the diameter of the holes in the photonic crystals grows by 2 nm, and this changes the optical path length by ~ 2 nm $\cdot$ (n_{Si} - n_{Air}) ~ 5 nm, which shows that spectral measurements are extremely sensitive to structural shifts. However, this is an upper bound because the cavity modes extend outside the membrane. To estimate this more precisely, we have simulated the L4/3 cavity discussed in Ref. [4], for which we find the shift in resonance wavelength per shift in silicon boundary to be -3.92 nm/nm, which is consistent with but more accurate than our simple estimate above. Thus, the data in Ref. [3] imply that the silicon edge is defined within a standard deviation of 1.1 nm/3.92 = 0.28 nm. This should be compared to the interatomic distance in silicon of 0.24 nm. This analysis builds on several unrealistically pessimistic assumptions: Other factors, such as the global shift, variations in wafer thickness, chemical composition at the edges, etc. all contribute to the experimental value found by Panuski *et al.*, which implies that the actual disorder is lower than our estimate. Also, we have considered the L4/3 cavity for which the standard deviation they measure is the largest. Therefore, the structural disorder is already today at the atomic scale in foundry production, so this implies that our method could be directly used to scale up the production of genuinely atomic-scale devices - even in a foundry where the resolution is limited to several tenths of nanometers.

In summary, our self-assembly method allows making features well below the resolution limit in both industry and research despite using only conventional fabrication techniques. This is directly evidenced by our devices showing gaps below 1 nm, which should be compared to the state of the art today in the "3 nm" node, namely 12 nm, or the "0.5 nm eq" node at the end of the industry roadmap in 2037, namely 8 nm.

S2. INVESTIGATION OF PULL-IN INSTABILITIES DUE TO SURFACE FORCES

S2.1. The effect of built-in stress release

The built-in stress in the device layer of silicon-on-insulator wafers causes expansion or contraction when the silicon oxide is selectively etched away to release the structures. This means that the initial gaps in the platforms we investigate, g_0, i.e., the gaps as they would be without the surface forces, do not exactly correspond to the fabricated gaps before underetching, g_f. The initial gap is modified by the stress-release displacement, Δg, which we correct for in our experiments as explained below. This displacement is experimentally obtained by including a reference platform with a fabricated 520 nm gap for each spring constant and platform width, which is large enough to diminish the surface forces by orders of magnitude [5]. We acquire scanning electron microscope (SEM) images of all such devices and perform image analysis to extract Δg. As mentioned in the Methods section of the main text, the platforms are equipped with scales on the sides, see Fig. S2a, allowing us to accurately extract their displacement from an SEM image acquired at high scan speed. The scales are analyzed by first using Sobel edge detection as shown in Fig. S2b, followed by a Radon transform as shown in Fig. S2c. The latter detects the angle of rotation of the device relative to the coordinate system of the image and compensates for it by counter-rotating with a bi-linear rotation. The pixel columns containing the scales are found by column-wise fast Fourier transforms and identification of the Fourier components matching the known periodicity of the scales as shown in Fig. S2d. The extracted pixel columns are then limited to the rows containing periodic structures, leaving only the areas marked in

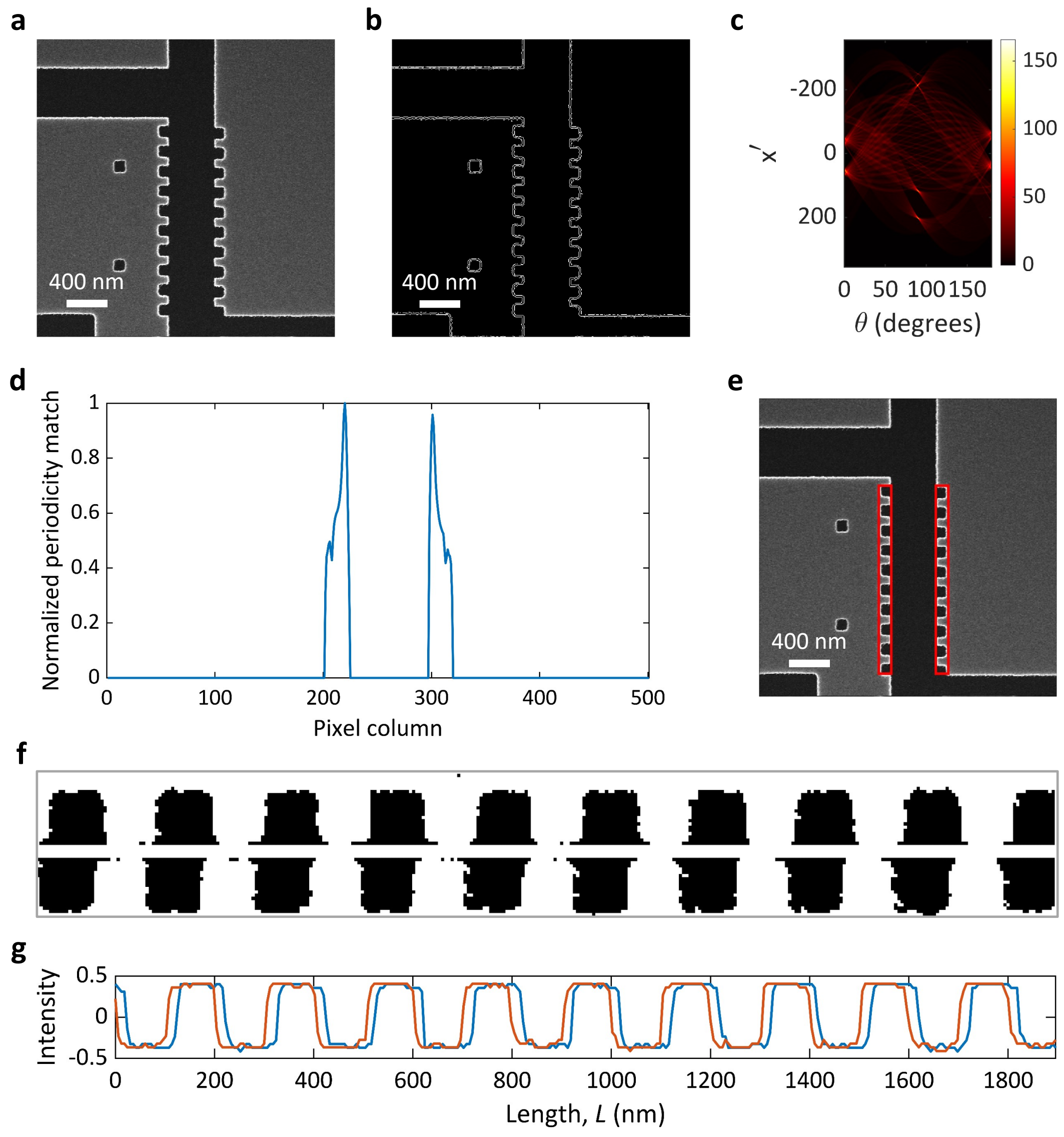

Supplementary Figure S2. **Automatic image-analysis of relative scale displacement.** **a**, Top-view SEM image of a scale attached to the platform with a fabricated gap, g_{f}, of 520 nm (after underetching). **b**, Sobel edge detection. **c**, Radon transform of the image to measure the rotation angle of the device relative to the image coordinate system. Here, Θ is the angle of rotation of an axis centered on the image and x' is the distance in pixels of a given pixel from the rotated axis. **d**, Match between periodicity found in the image columns and the known periodicity of the scales. **e**, Detected scales. **f**, Scales cropped and converted to a binary image. **g**, Graph obtained by summing the pixel counts of the black-and-white image, normalizing, and centering.

Fig. S2e. The detected scales are cropped from the image and converted to binary images

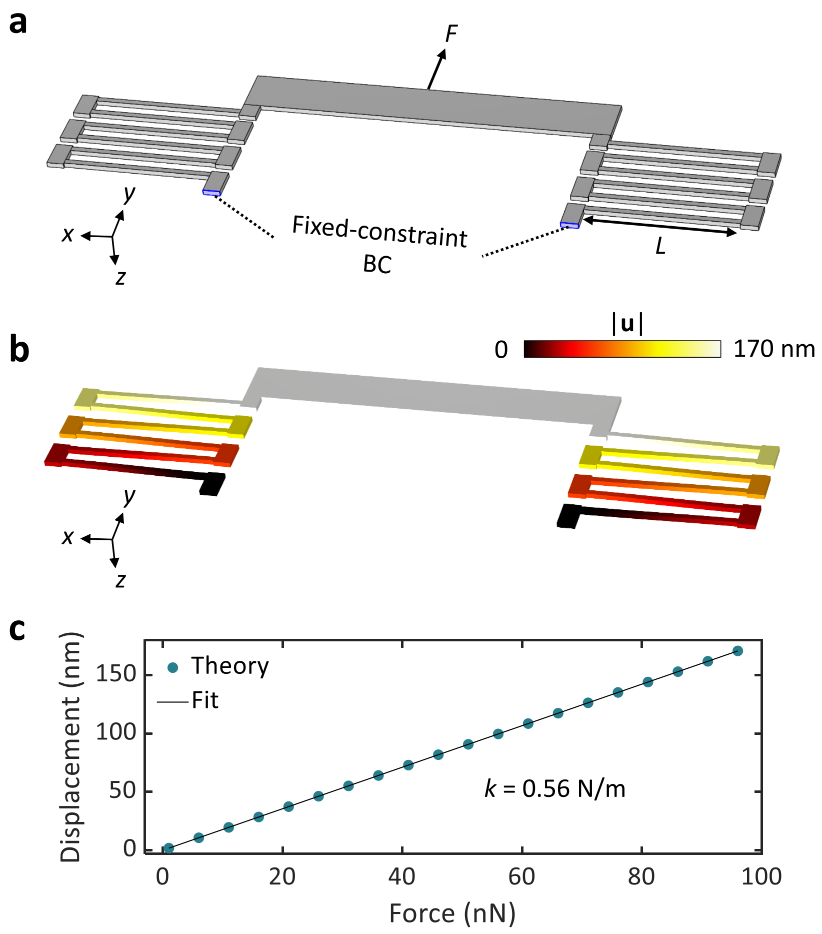

Supplementary Figure S3. **Numerical simulations for the extraction of spring constants, k.** **a**, Simulated geometry: A load, F, is applied at the silicon platform in the y-direction and the springs are anchored using the fixed-constraint boundary condition. The folded guided cantilever length is defined by the parameter L. **b**, The simulated displacement magnitude, $|\mathbf{u}|$, of the device with an applied load $F = 96$ nN. The displacement is exaggerated for illustration purposes. **c**, Force-displacement plot for the simulated device with cantilever length, $L = 6.4$ μm, a linear fit to which results in a spring constant of 0.56 N/m.

as seen in Fig. S2f, from which the number of black pixels in each column is counted to give the normalized plot seen in Fig. S2g. The phase difference between the two resulting curves is found by Fourier analysis. Based on measurements of 57 devices, we find a mean stress-release displacement of 19.4 nm with a standard deviation of 2.8 nm, independent of the platform width and the spring constant. Finally, the initial gap, g_0, used in Fig. 1d of the main text, is obtained by systematically acquiring SEM images of the platforms before

removing the buried oxide layer to extract the fabricated gap, g_f, followed by subtraction of the known displacement due to stress release, Δg, such that the initial gap is extracted as $g_0 = g_\mathrm{f} - \Delta g$.

S2.2. Numerical simulations for extracting the spring constant

The values of the spring constants are extracted by simulating their force-displacement curves in COMSOL Multiphysics using the full anisotropic elasticity matrix for silicon [6], the correct crystalline axis orientation of the silicon-on-insulator wafer, and the SEM-extracted geometries of the fabricated springs. Figure. S3a shows the geometry of the device used to extract the spring constant of folded guided cantilever spring systems, with the cantilever length denoted by L. The geometry consists of a silicon platform attached to two folded guided cantilevers, thus replicating the devices used in Fig. 1 of the main text. A load, F, is applied on the platform in the direction of the y-axis as shown by the arrow in Fig. S3a, and the springs are anchored using a fixed-constraint boundary condition. Figure. S3b shows the simulated displacement magnitude of the device with $L = 6.4$ μm at an applied load of $F = 96$ nN. The force-displacement curve is plotted in Fig. S3c and a spring constant of k $= 0.56$ N/m is extracted via a linear fit. We note that our simulations include geometric non-linearity, but the force-displacement relation is highly linear, so we conclude that the springs in our experiments are linear within a very wide displacement range. The spring constants used in the experimental work are extracted by repeating this process for varying cantilever lengths L.

S2.3. Mapping the threshold for self-assembly by surface forces

We fabricate a total of 2688 devices distributed across two samples (Sample A: 1536 devices; Sample B: 1152 devices) with different values of platform width, w, fabricated gap, g_f, and spring constant, k, as discussed in the Methods section. We characterize the devices using SEM performed with a single high-speed scan to minimize charge-induced displacement of the platforms, except when imaging for illustrative purposes, as in Fig. 1c in the main

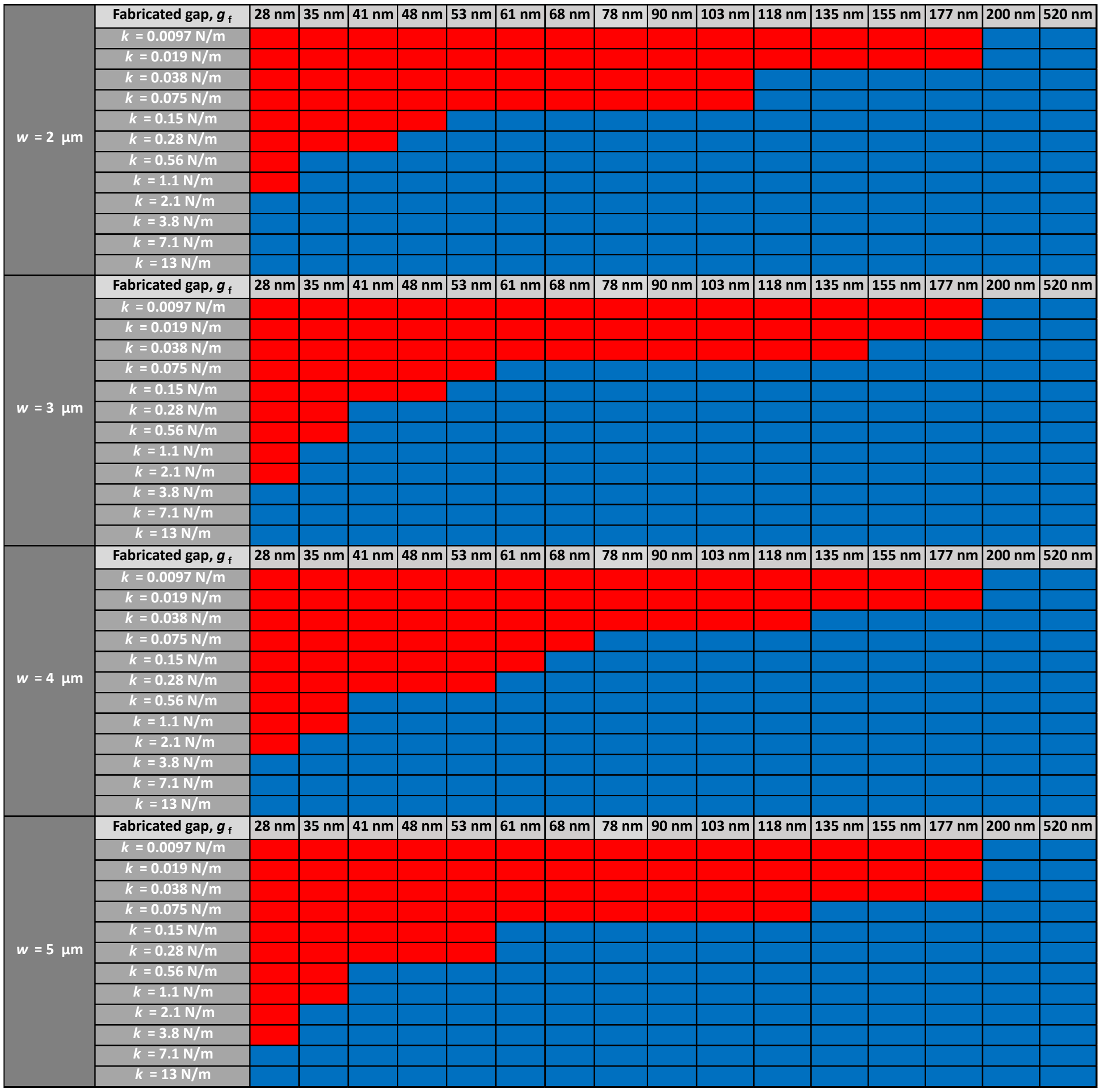

Supplementary Figure S4. **Post-underetching structural state of silicon platforms with widths 2, 3, 4, and 5 µm, respectively, for Sample A.** The columns indicate platforms with a fabricated gap, g_{f}, measured before releasing the structures, and the rows indicate platforms with different spring constants, k. Each block shows the experimental data for a specific platform width, w. The red cells represent platforms that collapsed in-plane on the anchored silicon, and the blue cells indicate platforms that did not collapse.

text. Figures S4 and S5 show the resulting map of the devices that either collapsed in-plane (red) on the anchored silicon or did not collapse (blue). The data set in Figs. S4 and S5 is used to plot Fig. S8 and Fig. 1d of the main text. No data has been discarded in these figures but the 11 devices that failed due to fabrication imperfections or spring failure, such

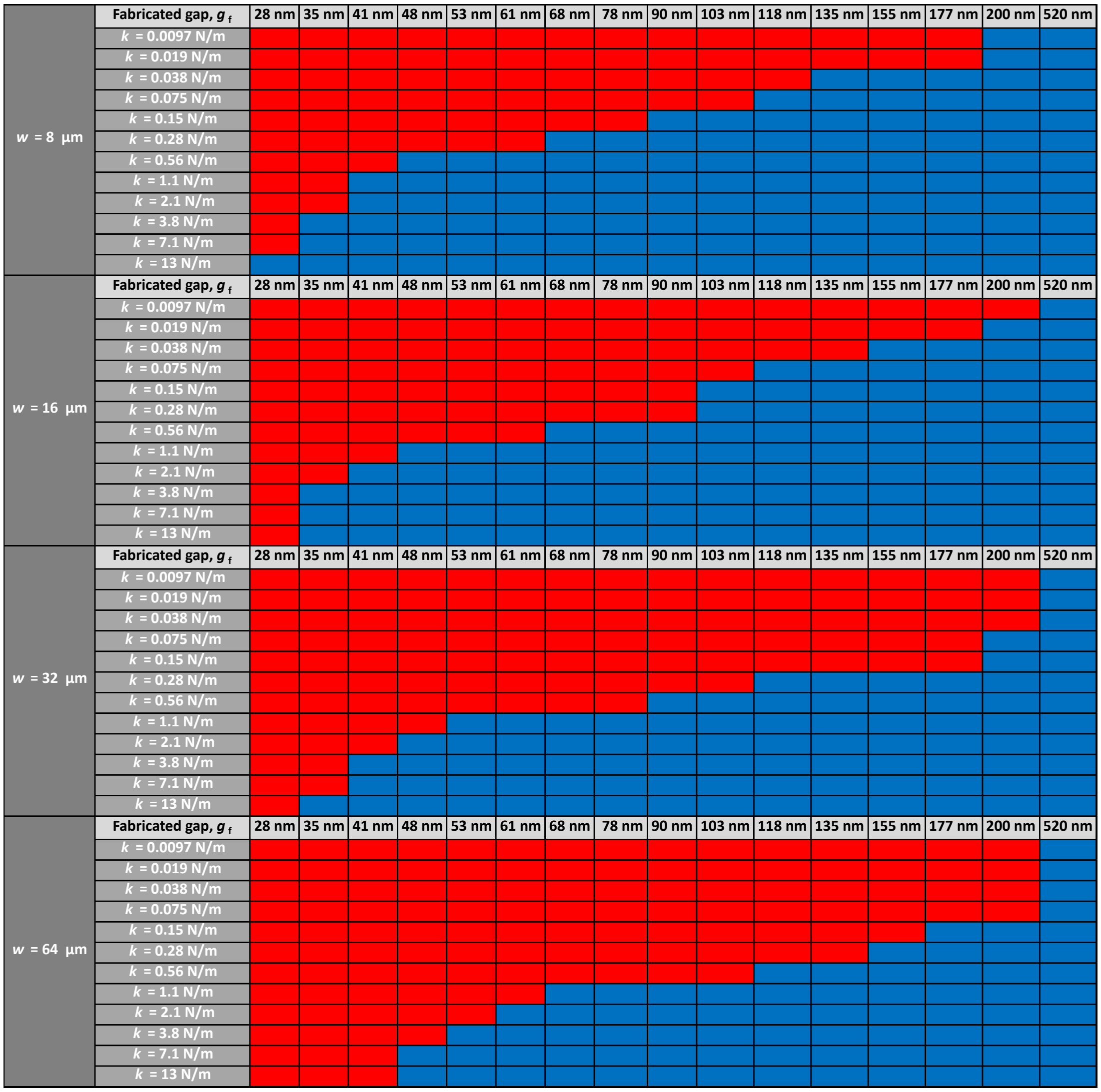

Supplementary Figure S5. **Post-underetching structural state of silicon platforms with widths 8, 16, 32, and 64 μm, respectively, for Sample A.** The columns indicate platforms with a fabricated gap, g_f, measured before releasing the structures, and the rows indicate platforms with different spring constants, k. Each block shows the experimental data for a specific platform width, w. The red cells represent platforms that collapsed in-plane on the anchored silicon, and the blue cells indicate platforms that did not collapse.

as out-of-plane collapse (yellow), are excluded in the following.

Figure. S8 illustrates the experimental data set for all platform widths on a single plot: green-filled and purple-filled circles depict the collapsed and not-collapsed devices, respectively, on a log-log plot with the vertical axis showing the initial gap, g_0, of the devices

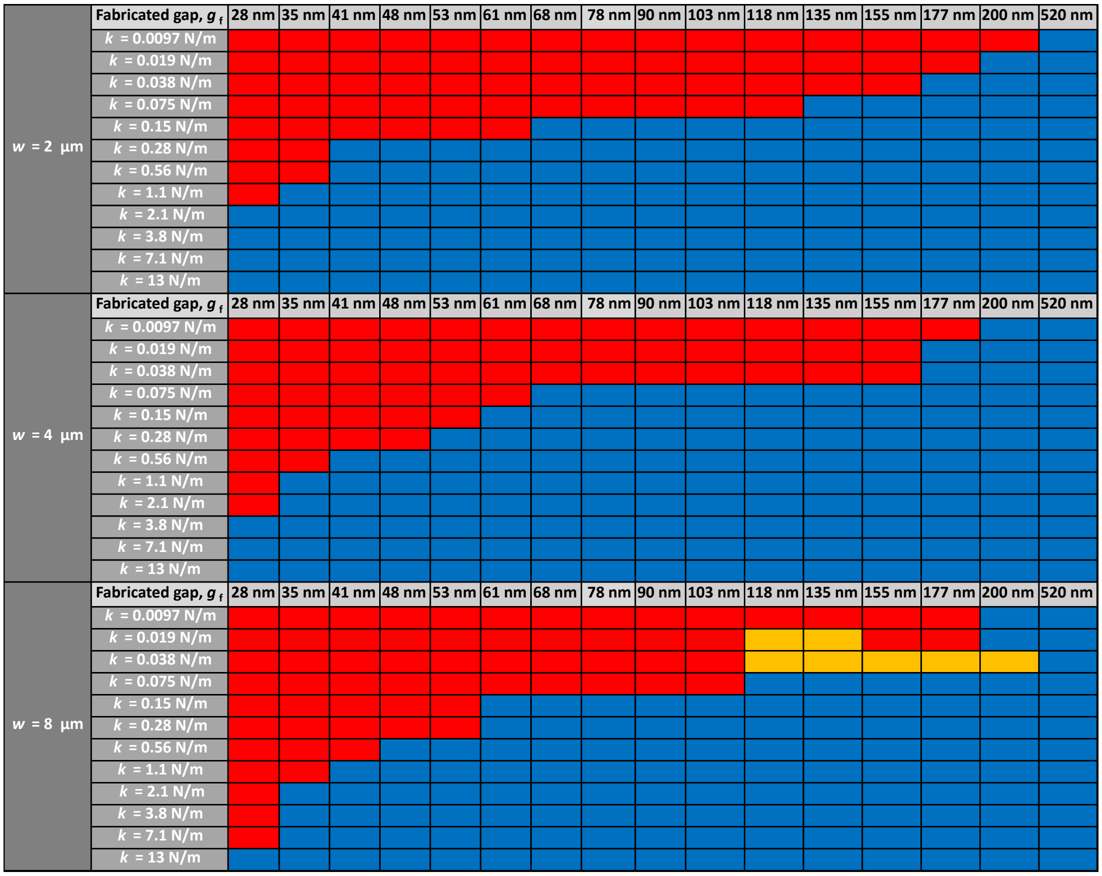

Supplementary Figure S6. Post-underetching structural state of silicon platforms with widths 2, 4, and 8 μm, respectively, for Sample B. The columns indicate platforms with a fabricated gap, g_f, measured before releasing the structures, and the rows indicate platforms with different spring constants, k. Each block shows the experimental data for a specific platform width, w. The red cells represent platforms that collapsed in-plane on the anchored silicon, and the blue cells indicate platforms that did not collapse. The yellow cells indicate the devices excluded due to fabrication imperfections or spring failure.

and the horizontal axis showing the ratio of the spring constant to the platform area, k/A. The circle size represents the size of the platforms. For fixed values of w and k, we identify two gaps: the largest value of g_0 for which the collapse occurs (dark green filled circle) and the smallest value of g_0 for which the collapse does not occur (dark purple filled circle). Using these gaps, we find that all platforms for which $g_0 < 3.8(k/A)^{-0.55}$ collapse (upper bound) and all platforms for which $g_0 > 16.8(k/A)^{-0.54}$ do not collapse (lower bound). A simplified version of Fig. S8 is shown as Fig. 1d in the main text, where the platform sizes

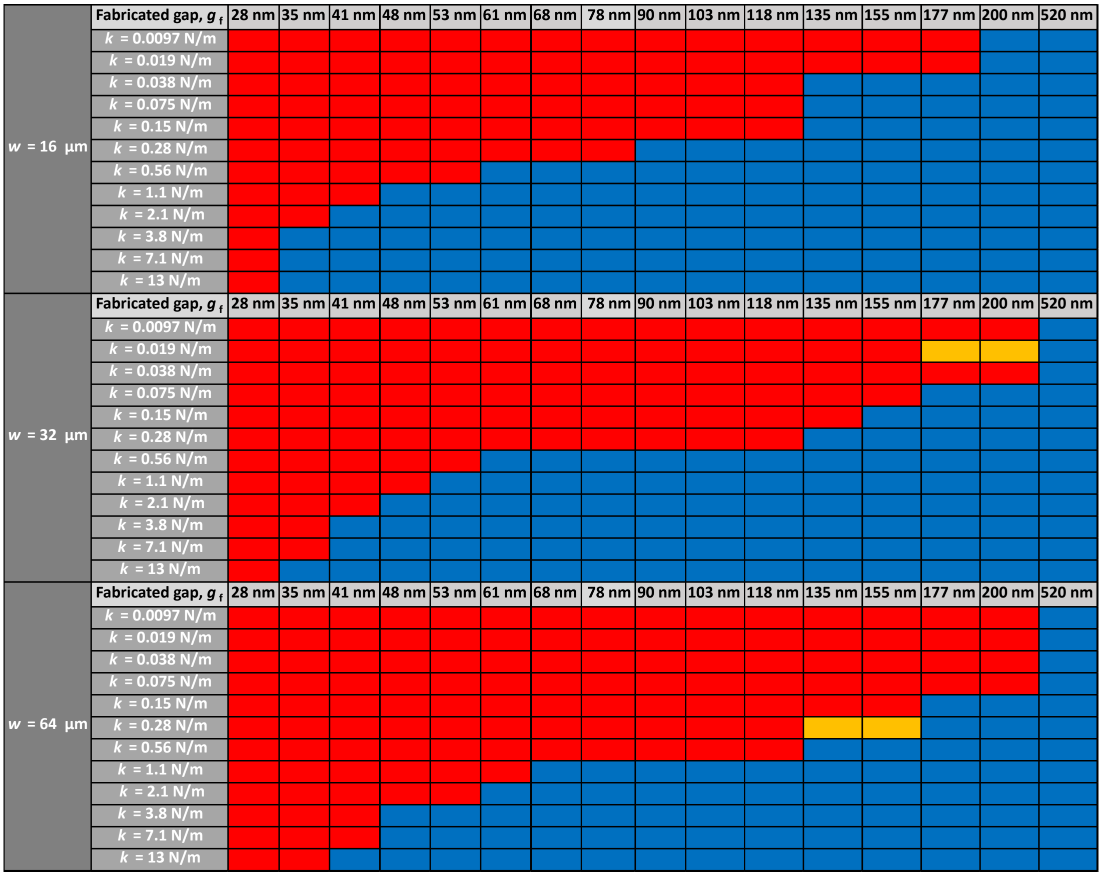

Supplementary Figure S7. **Post-underetching structural state of silicon platforms with widths 16, 32, and 64 μm, respectively, for Sample B.** The columns indicate platforms with a fabricated gap, g_{f}, measured before releasing the structures, and the rows indicate platforms with different spring constants, k. Each block shows the experimental data for a specific platform width, w. The red cells represent platforms that collapsed in-plane on the anchored silicon, and the blue cells indicate platforms that did not collapse. The yellow cells indicate the devices excluded due to fabrication imperfections or spring failure.

are illustrated with the same circle size, and only a single color scheme is used to display the collapsed and non-collapsed states of the devices.

The experiment is repeated on Sample B to verify the reproducibility and robustness of our approach. The raw data is shown in Figs. S6 and S7. Figure S9 presents the self-assembly design space with compliant silicon structures for Sample B (1152 devices). In this case, we find that all platforms for which $g_0 < 4.2(k/A)^{-0.48}$ collapse (upper bound) and all platforms for which $g_0 > 24(k/A)^{-0.47}$ do not collapse (lower bound). We attribute the

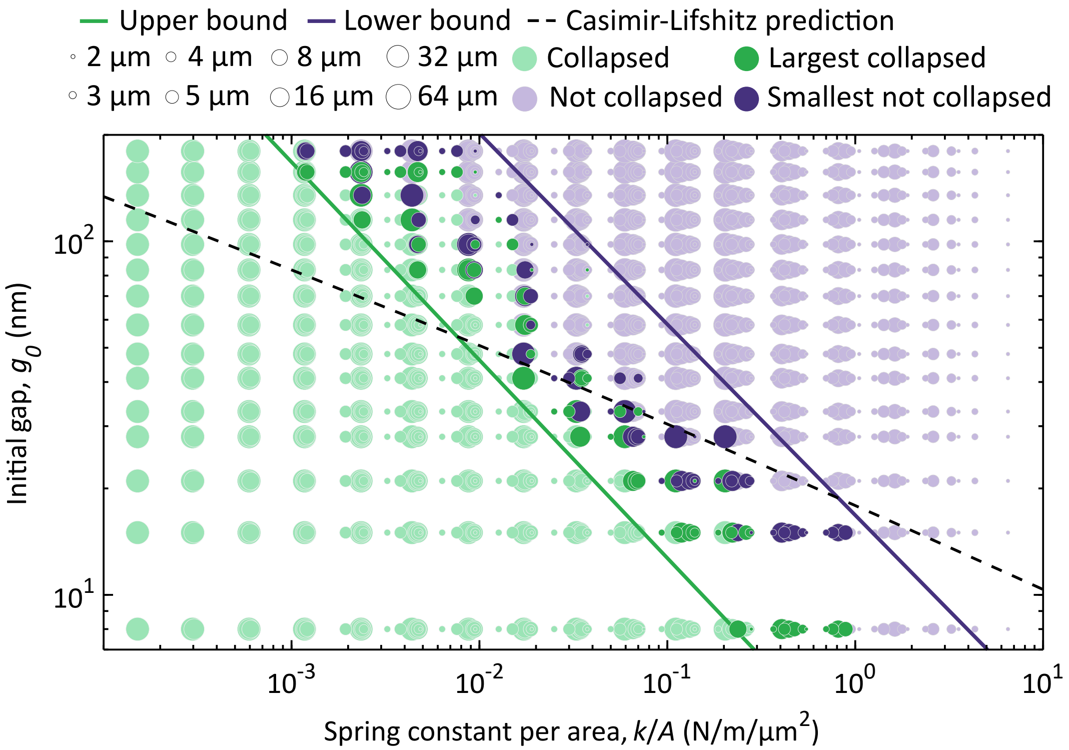

Supplementary Figure S8. Measured map of the design space for self-assembly with compliant silicon structures (Sample A). The map is obtained by characterizing 1536 platforms by SEM. The different sizes of the circles represent the different widths of the platforms. The dark purple circle indicate the smallest initial gap not leading to a collapsed platform, and the dark green circle indicates the largest initial gap leading to a collapsed platform. All the devices below the upper bound collapse, while those above the lower bound do not. The figure is identical to Fig. 1d in the main text except that here we also show the dependence on platform width.

small differences in bounds between samples A and B to the fabrication variations across the sample due to proximity effects and etching (see Section S4.2). Despite these differences, the regions for using or avoiding the directed collapses span several orders of magnitude in the phase-space parameters.

S2.4. The critical gap of Casimir-Lifshitz theory as the threshold for self-assembly by surface forces

The van der Waals force, which is responsible for surfaces adhering when they touch, is the short-distance (non-retarded) limit of the more general Casimir-Lifshitz force, which,

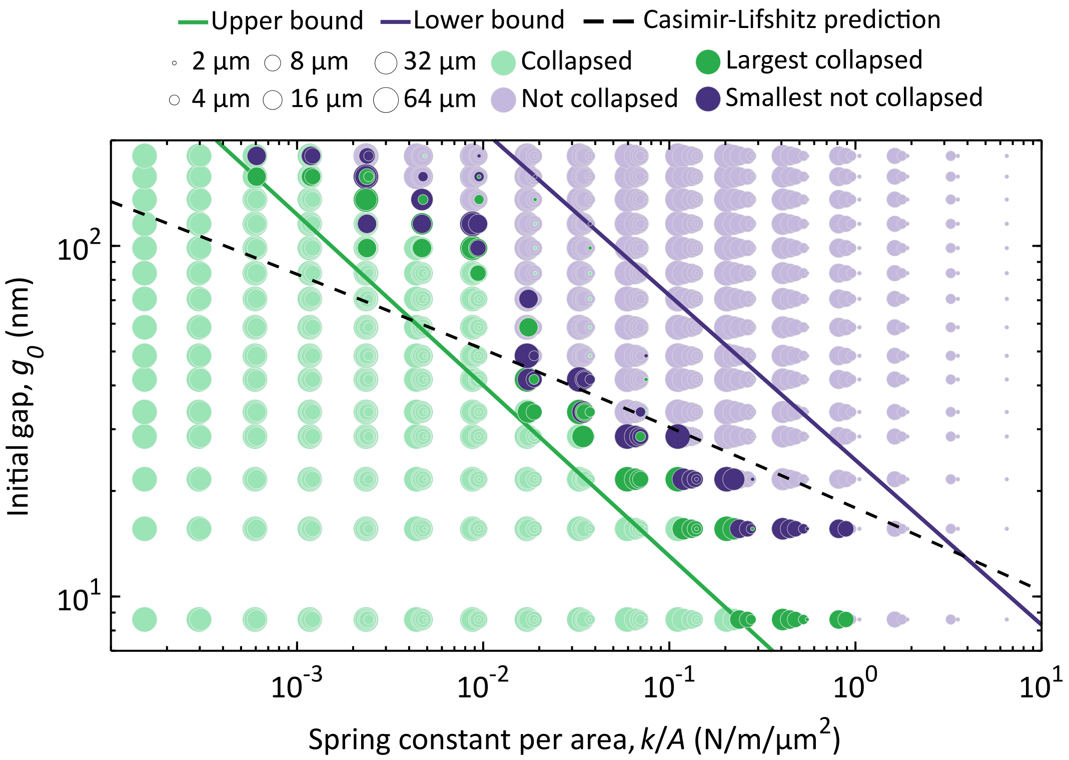

Supplementary Figure S9. **Measured map of the design space for self-assembly with compliant silicon structures (Samples B).** The map is obtained by characterizing 1152 platforms by SEM. The different sizes of the circles represent the different widths of the platforms. The dark purple circle indicate the smallest initial gap not leading to a collapsed platform, and the dark green circle indicates the largest initial gap leading to a collapsed platform. All the devices below the upper bound collapse, while those above the lower bound do not.

in the idealized case of perfectly reflecting infinitely extended surfaces reduces to the long-range attractive force described by Casimir [7]. When the intervening material between two surfaces is a vacuum, the Casimir-Lifshitz force is attractive and increases non-linearly with decreasing separation, g, between the surfaces. Suppose two surfaces are initially separated by the critical gap, g_c. In that case, the Casimir-Lifshitz force can overwhelm the elastic forces that hold the surfaces in place, bring them in contact, and subsequently stick them onto each other. We use a lumped-element model describing the elastic force by a linear spring with spring constant k to calculate the critical gap. To approximate the interaction force, we use the additive approximation known as the proximity-force approximation (PFA) in which the interacting bodies are divided into infinitesimal parallel plates, whose energy is integrated to extract the total interaction energy [8]. The Casimir force can then be

extracted by taking the derivative of the energy with respect to the displacement [9].

The total force between two parallel surfaces separated by a gap $g = g_0 - x$, where g_0 is the initial gap between the surfaces and x is their displacement due to the Casimir-Lifshitz attraction, is given by

$$F_{\text{tot}}(g) = AF_{\text{CL}}(g) - k(g_0 - g). \tag{S1}$$

Here, A is the area and $F_{\text{CL}}(g)$ is the Casimir-Lifshitz force per unit area between two surfaces given by [5]

$$\begin{aligned} F_{\text{CL}}(g) = -\frac{\hbar}{2\pi^2 c^3}\int_1^\infty p^2 \mathrm{d}p \int_0^\infty \xi^3 \mathrm{d}\xi \Bigg\{ \left[\left(\frac{K + \epsilon(i\xi)\, p}{K - \epsilon(i\xi)\, p} \right)^2 e^{2(\xi/c)pg} - 1 \right]^{-1} \\ + \left[\left(\frac{K + p}{K - p} \right)^2 e^{2(\xi/c)pg} - 1 \right]^{-1} \Bigg\}, \end{aligned} \tag{S2}$$

with $K = \sqrt{p^2 - 1 + \epsilon(i\xi)}$ and the dielectric function of silicon at imaginary frequencies given by $\epsilon(i\xi) = 1 + \frac{10.703}{1 + (\xi * 1.506 * 10^{-16})^{1.83}}$, where ξ is measured in rad/s [10]. The system loses linear stability at the point g^* when

$$\frac{\partial F_{\text{tot}}(g^*)}{\partial g} = 0. \tag{S3}$$

The critical gap, g_c, is defined as the initial gap that leads to the instability point, g^*, after the surfaces have been attracted by the Casimir force, i.e.,

$$g_c = \frac{A}{k} F_{\text{CL}}(g^*) + g^*. \tag{S4}$$

To calculate the critical gap, g_c, for a given k/A, we first solve Eq. (S3) numerically for g^*, which we subsequently insert in Eq. (S4). The resulting g_c is included in Fig. 1d in the main text and Figs. S8 and S9. The agreement is only good in the range where the PFA is expected to be valid, and besides the possible sources of deviations discussed in the main text, it should be pointed out that systematic errors in SEM measurements of few-nanometer features can be significant.

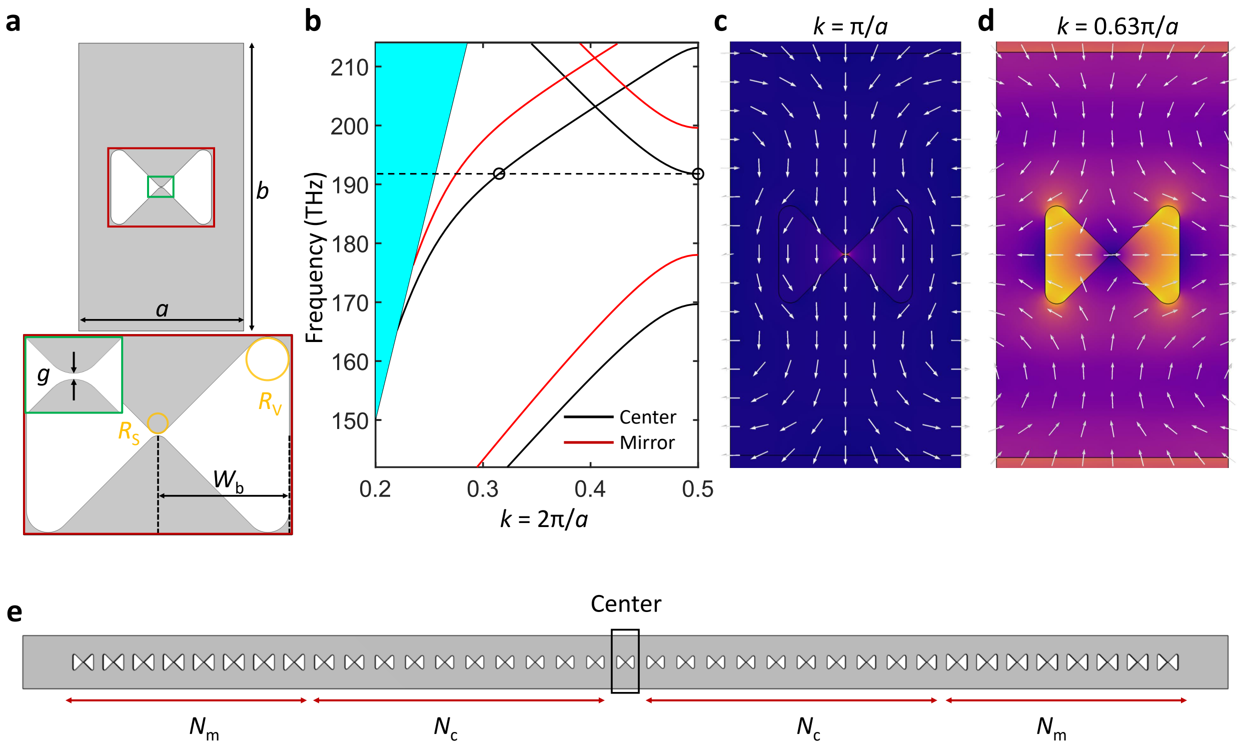

Supplementary Figure S10. Design of a photonic-crystal nanobeam bowtie cavity. a, Geometric parameters of a single bowtie unit cell with an air bowtie of width g. **b**, Band structure (only quasi-transverse-electric modes) for the central unit cell (W_b = 116 nm, black curve), and the mirror unit cell (W_b = 139 nm, red curve). The blue region indicates the light cone. **c,d**, normalized $|\mathbf{E}|$-field of the center bowtie unit cell in the middle plane of the structure and at the edge of the Brillouin zone (**c**), and at a different wavenumber but the same frequency (**d**). The white arrows indicate the electric-field direction at the center of the silicon slab. **e**, Illustration of the photonic-crystal nanobeam bowtie cavity comprised of a central unit cell, tapering unit cells, N_c, and mirror unit cells, N_m.

S3. DESIGN OF NANOBEAM PHOTONIC-CRYSTAL CAVITIES WITH BOWTIE UNIT CELLS

S3.1. Band structures and cavity design

The nanobeam photonic-crystal cavities we explore for self-assembly have a unit cell with a single-digit nanometer bowtie width at the center. Figure S10a shows a triangular bowtie unit cell with a nanobeam width of b = 700 nm, a lattice constant of a = 400 nm, an

air-bowtie width of $g = 2$ nm, and a triangle width of $W_\mathrm{b} = 116$ nm. The bowtie angle is fixed to 90°. The measured fabrication constraints, i.e., the smallest solid ($R_\mathrm{S} = 10$ nm) and void ($R_\mathrm{V} = 20$ nm) radii of curvature, are included in the cavity design to comply with our fabrication process, as shown in Fig. S10a and discussed in Section S4.2. The band diagram of the unit cell is computed with a finite-element method using COMSOL Multiphysics and shown with solid black lines in Fig. S10b, indicating that the second-order mode at the Brillouin-zone edge is a bowtie mode (Fig. S10c). Figure S10b also includes the band structure for a unit cell with slightly modified parameters ($b = 700$ nm, $a = 400$ nm, $g = 2$ nm, $W_\mathrm{b} = 139$ nm), which we use as the mirror unit cell of our nanobeam cavity. We observe that the bowtie band at the edge of the Brillouin zone resides within the band gap of the mirror unit cell, which allows the generation of spatially confined cavity modes by adiabatic tapering of the mirror unit cell into the center unit cell and back into the mirror unit cell. The band diagrams show that another mode (Fig. S10d) is present at the same energy but at a lower wavenumber. These two modes, as shown with the superimposed vector fields in Figs. S10c and d, have different symmetries, which ensures that a cavity design respecting the symmetry cannot couple the bowtie mode to the other modes. The exact cavity geometry is built following a well-known procedure [11] in which the unit cell is continuously transformed along N_c unit cells in both directions from the center to the mirror unit cell, followed by N_m mirror unit-cell sections. Figure S10e shows the cavity geometry used for the waveguide-coupled photonic-crystal nanobeam cavities, which uses $N_\mathrm{c} = 10$ and $N_\mathrm{m} = 8$. Table. S1 shows the geometric parameters for the waveguide-coupled photonic-crystal nanobeam cavity, notably the value of W_b at the i-th defect unit cell, $W_{\mathrm{b,\,i}}$. On the contrary, the cavity geometry used for the resonant scattering measurements of Fig. 3 in the main text uses a much shorter defect region with $N_\mathrm{c} = 3$ and $N_\mathrm{m} = 16$ and Table. S2 shows the geometric parameters for that cavity. The short defect region ensures the presence of measurable out-of-plane scattering, thereby enabling resonant scattering measurements.

Parameter	Dimensions (nm)
Nanobeam width, b	700
Lattice constant, a	400
Air-bowtie width, g	2
$W_{\mathrm{b},1}$	116
$W_{\mathrm{b},2}$	116
$W_{\mathrm{b},3}$	117
$W_{\mathrm{b},4}$	117
$W_{\mathrm{b},5}$	118
$W_{\mathrm{b},6}$	119
$W_{\mathrm{b},7}$	120
$W_{\mathrm{b},8}$	122
$W_{\mathrm{b},9}$	124
$W_{\mathrm{b},10}$	126
$W_{\mathrm{b},11}$	130
$W_{\mathrm{b},12}$ - $W_{\mathrm{b},19}$	139

TABLE S1. Geometric parameters for the waveguide-coupled photonic-crystal bowtie nanobeam cavity.

S3.2. Evaluation of the effective mode volume

In this manuscript, the effective mode volume, V, of the explored cavities is evaluated by using the quasi-normal mode,

$$\frac{1}{V} = \mathrm{Re}\left[\frac{\epsilon_r\left(\mathbf{r}_0\right)\mathbf{E}\left(\mathbf{r}_0\right)\cdot\mathbf{E}\left(\mathbf{r}_0\right)}{\int_{V_T}\epsilon_r(\mathbf{r})\mathbf{E}(\mathbf{r})\cdot\mathbf{E}(\mathbf{r})\;dV + i\frac{c\sqrt{\epsilon_r}}{2\omega}\int_S \mathbf{E}\left(\mathbf{r}\right)\cdot\mathbf{E}\left(\mathbf{r}\right)\;dA}\right] \tag{S5}$$

where $\mathbf{r_0}$ is the position where mode volume is evaluated, $\mathbf{E}(\mathbf{r})$ is the electric field of the quasi-normal mode, $\epsilon_\mathbf{r}$ is the dielectric constant, the volume integral is taken over the entire simulation domain, V_T, and the surface integral is over the surfaces implementing the radiation boundary condition, S [12]. We select $\mathbf{r_0}$ to be the center of the nanocavity, which we define as the center of the central bowtie (at half the bowtie width and in the mid-plane of the silicon device layer). Evaluating at the center point is a robust method for calculating

Parameter	Dimensions (nm)
Nanobeam width, b	700
Lattice constant, a	400
Air-bowtie width, g	2
$W_{b,1}$	118
$W_{b,2}$	119
$W_{b,3}$	121
$W_{b,4}$	126
$W_{b,5}$ - W_{b20}	140

TABLE S2. Geometric parameters for the photonic-crystal bowtie nanobeam cavity for the resonant scattering measurements.

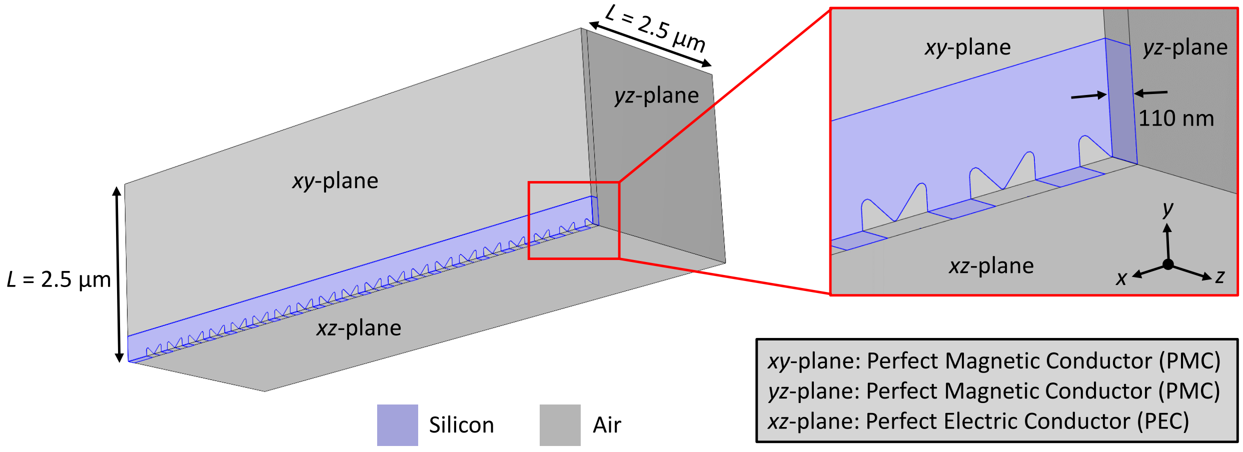

Supplementary Figure S11. Simulation domain and boundary conditions for the nanobeam cavity. The numerical simulation domain and boundary conditions used for simulating the cavity, which exploit the symmetry of the nanobeam cavity. The blue shaded region is the silicon nanobeam, and the grey region is the bowtie air holes and the surrounding air-box.

the mode volume because it is unaffected by the lightning-rod surface fields that appear at surfaces due to the electromagnetic boundary conditions [13, 14].

S3.3. Numerical calculations and mesh convergence of nanobeam bowtie cavities.

In our manuscript, we model nanobeam bowtie cavities using a finite-element method (FEM) and a complex eigensolver in COMSOL Multiphysics, which allow us to find the quasi-normal modes supported by the cavity. An illustration of the implemented numerical model is shown in Fig S11. We simulate one-eighth of the nanocavity geometry (except for the disorder simulations discussed in Section S3.5) as allowed by the symmetry of the nanocavity geometry and of the fundamental mode of interest. The symmetry of the latter is selected by setting the appropriate boundary conditions. In order to accurately capture the underlying physics, these calculations require extremely fine meshes in the bowtie region for single-nanometer bowtie widths. Therefore, simulating one-eighth of the nanocavity structure is beneficial to reduce memory requirements and computation time. In Fig S11, the blue region is the silicon, and the grey region is the air. Perfect magnetic conductor (PMC) boundary conditions are applied at the xy- and yz-planes. A perfect electric conductor (PEC) boundary condition is used at the xz-plane. The air box surrounding the nanobeam cavity has a height and depth of $L = 2.5$ μm. The length of the air box surrounding is equivalent to that of the nanobeam cavity. First-order scattering boundary conditions are used on the air box surrounding to model the radiation boundary condition at infinity.

Geometric Entity	Maximum element size [nm]	Minimum element size [nm]	Maximum element growth rate
Silicon nanobeam	$\lambda_r/n_{Si}/m$	$\lambda_r/n_{Si}/5/m$	1.6
Boundary 1	$5/m$	$0.5/m$	1.4
Boundary 2	$10/m$	$1/m$	1.4
Edge	$10/m$	$1/m$	1.4
Air box surrounding	$\lambda_r/n_{Air}/m$	$\lambda_r/n_{Air}/5/m$	1.7

TABLE S3. Mesh element sizes for different geometric entities of the nanocavity geometry. The sizes in the different regions are parametrized with variable m, which we vary to study numerical convergence. The value used for the reference wavelength,λ_r, and the refractive indices of silicon and air, n_{Si} and n_{Air}, are 1520 nm, 3.48 and 1, respectively.

The simulation domain is meshed with different resolutions or mesh-element sizes in

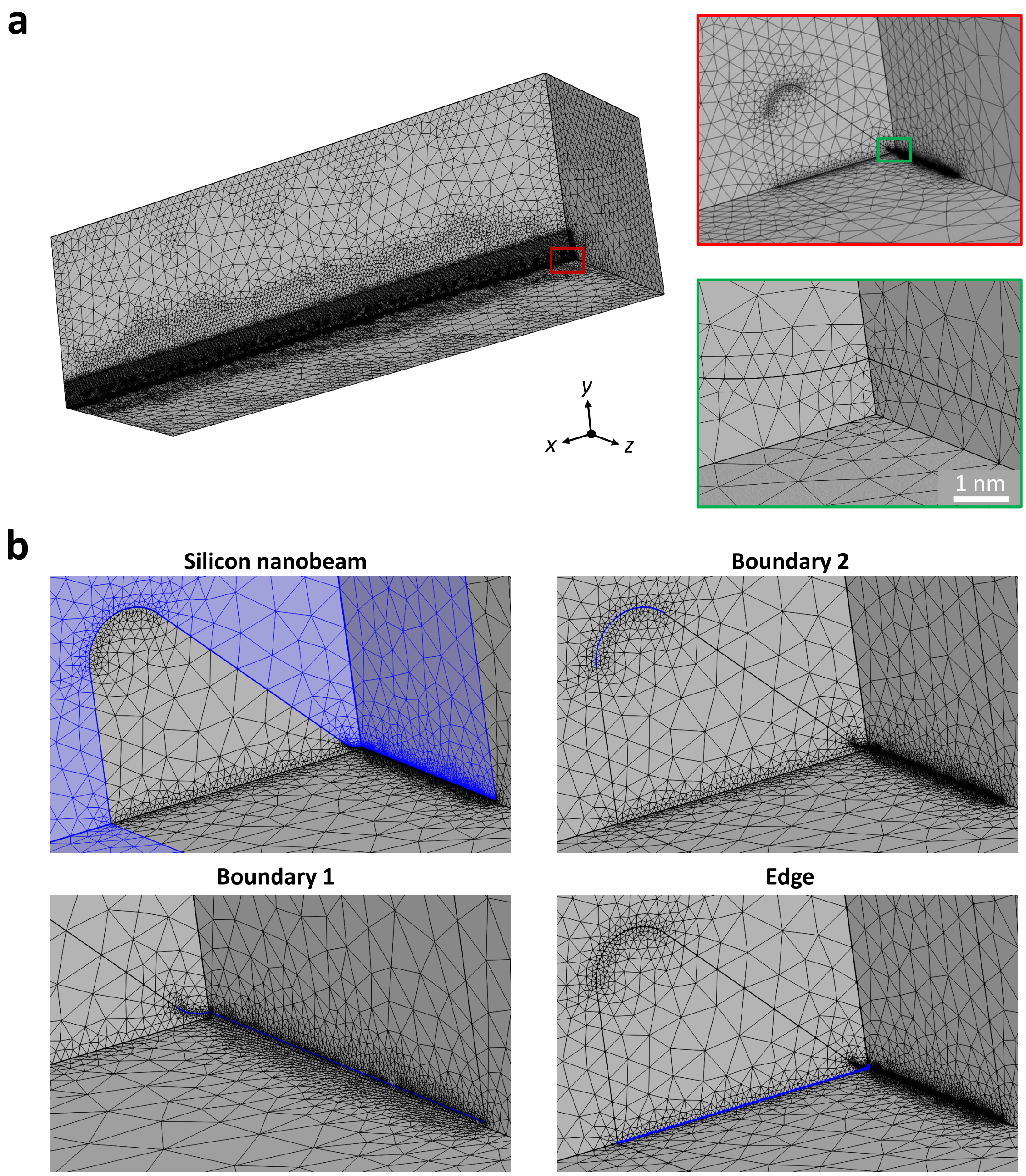

Supplementary Figure S12. **Mesh elements for the bowtie nanobeam cavity.** **a**, Mesh elements for one-eighth of the nanocavity geometry with the converged value of the mesh parameter, $m = 5$. **b**, Mesh elements for different nanobeam cavity geometry domains, boundaries, and edges. The blue region represents the region of interest.

different regions, e.g., the silicon nanobeam region, nanobeam air holes, air box, etc. The boundaries and edges around the bowtie region are meshed prior to the rest of the geometry and with a higher resolution and a smaller value of the element growth rate than the rest of

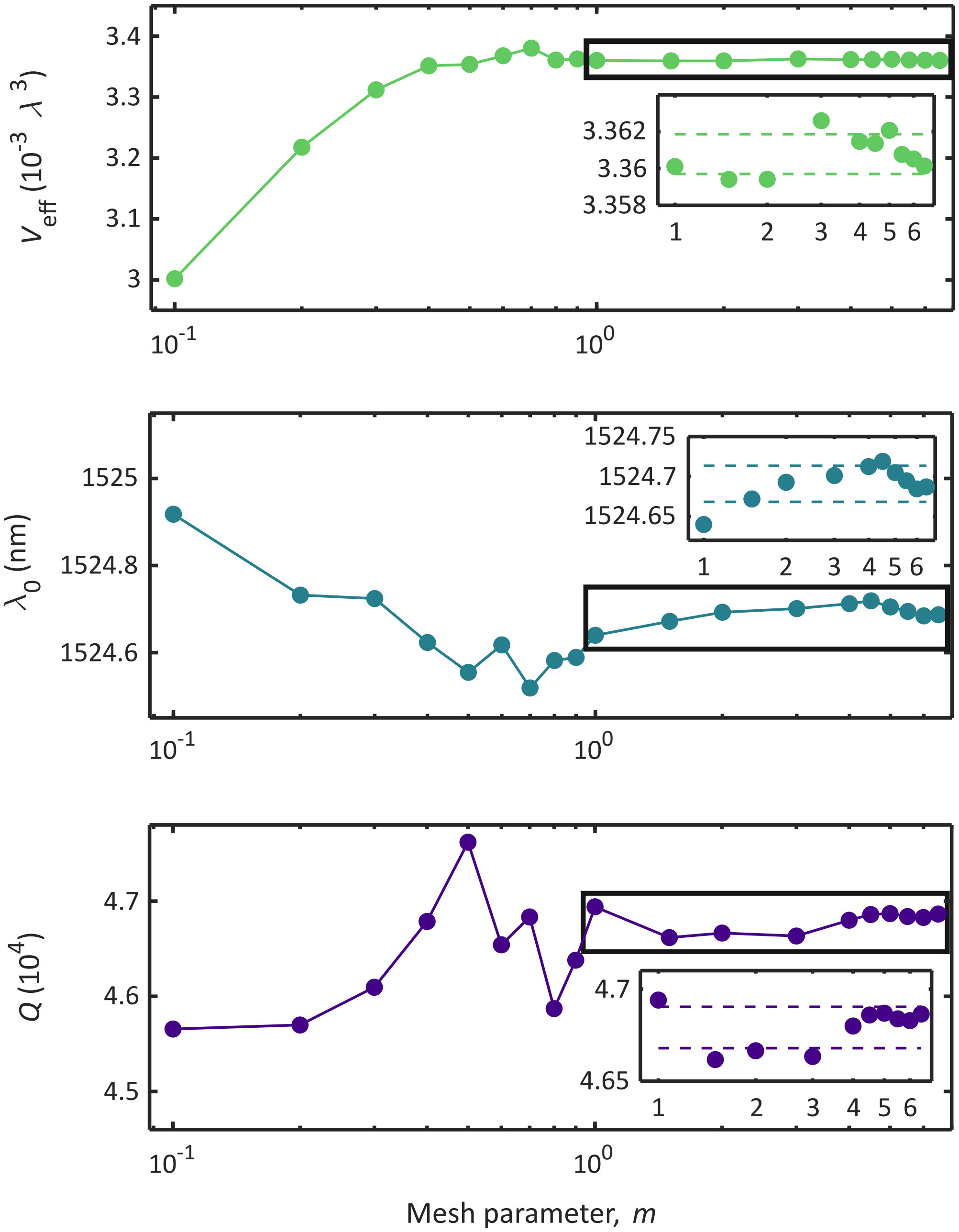

Supplementary Figure S13. **Mesh convergence test for a nanobeam bowtie cavity.** Convergence test and converged values for the mode volume, resonance wavelength, and quality factor using different values of the mesh parameter, m, plotted on a log scale. The insets show the log-scale plot of the regions highlighted with a black rectangular outline. The dashed lines represent the upper and lower bound of the standard deviation for the values in the highlighted region.

the structure in order to avoid discontinuities at the polygon vertices. The precise COMSOL

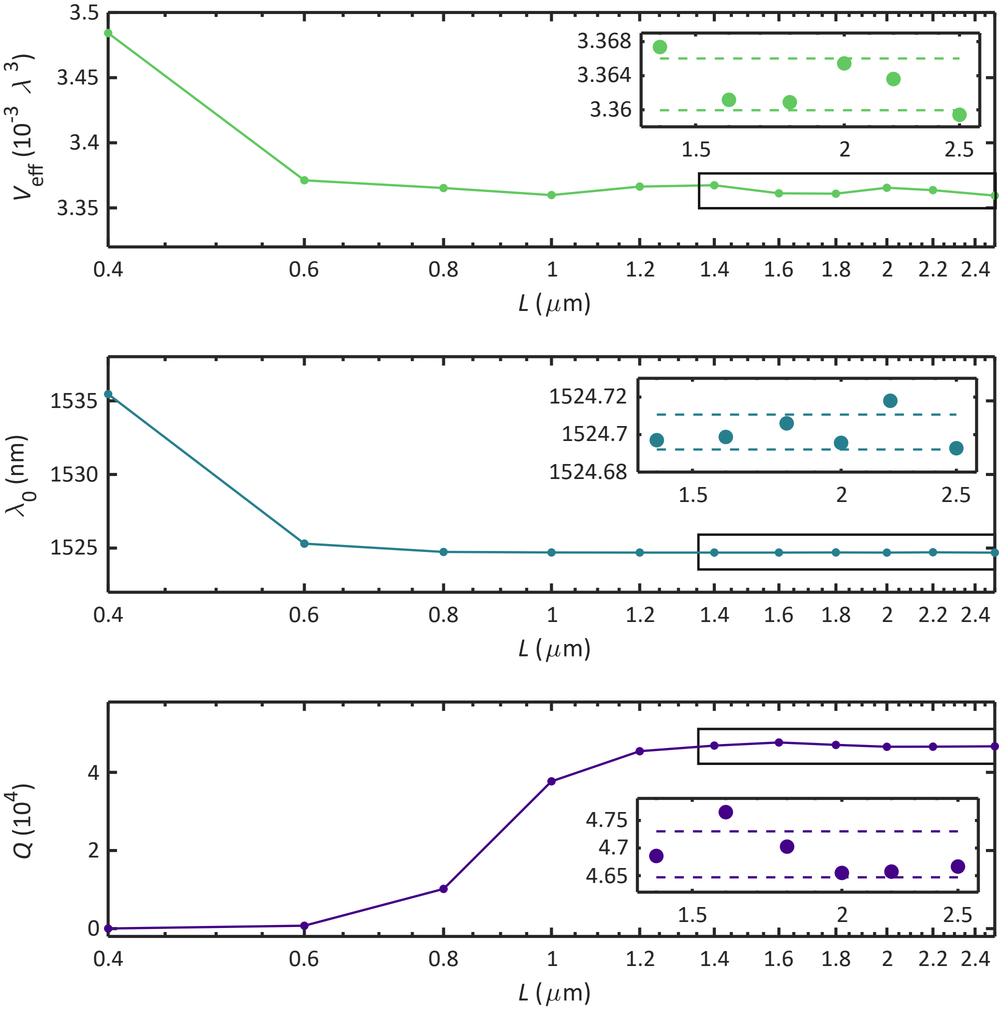

Supplementary Figure S14. Convergence test for air-box surrounding dimensions. Convergence test for the air-box surroundings and converged values for mode volume, resonance wavelength, and quality factor plotted on a log scale. The air-box height and depth represented by the parameter L are varied to perform the convergence test. The convergence test was performed with a mesh parameter of $m = 2$. The insets illustrate the log-scale plot of the regions highlighted with a black rectangular outline. The dashed lines represent the upper and lower bound of the standard deviation for the values in the highlighted region.

settings for the mesh in the regions specified in Fig. S12 are summarized in Table S3, all of them parameterized by a variable, m, which we vary to study the convergence of the fundamental cavity mode parameters. The resulting mesh for $m = 5$, which is the finest mesh we explore, is illustrated in Fig. S12. Figure S13 shows the mesh convergence results for the nanobeam cavity with the design parameters equivalent to the cavity shown in Fig. 2

in the main text. The mode volume, resonance wavelength, and quality factor are plotted for varying m, with the axis of the latter on a log scale. The insets show the plots for m values greater or equal to 1, with the dashed lines illustrating the upper and lower bounds of the standard deviation of the values in the highlighted region. For our model, the mesh converges around $m = 1$, and we attribute the deviations found for larger values of m to numerical noise, as most of the values fall within the bounds defined by the standard deviation. The numerical results reported in this work are all obtained from simulations that use a mesh parameter m within the converged range, typically $m = 1$, for which a simulation of a single quasi-normal mode (the one of interest) takes approximately 1.5 hours to run and consumes 150 GB of memory on an AMD EPYC 7702P 64-Core server.

In addition to testing the convergence of the cavity-mode parameters with respect to the size of the mesh, we also performed a convergence test on the air box surrounding the nanobeam. This is critical to ensure convergence of the calculated quality factors because too small surrounding air boxes may lead to large evanescent fields at the boundary that is supposed to mimic the radiation condition. Figure S14 shows the convergence test for the air box performed with $m = 1$, where the parameter L defines the height and depth of the air box as shown in Fig. S11. The cavity parameters converge for a surrounding air box of $L = 1.4$ μm, as most values are within the bounds defined by the standard deviation (same criteria as for the mesh size). We have used $L = 2.5$μm throughout the manuscript to model our cavities, which is well within the converged range.

S3.4. The role of the native oxide

The simulations in Sections S3.1 and S3.3, which employ an idealized air-bowtie nanobeam structure, do not consider the spontaneously formed native oxide layer in the air-exposed boundaries of the fabricated structures. Through high-resolution scanning transmission electron microscopy (STEM) and electron energy-loss spectroscopy (EELS), we measure the thickness of such native oxide layer (at the bowtie tips) to be between 2 and 2.5 nm (see Section S4.4) in good agreement with previous experimental observations [15] and ab-initio calculations [16]. The native oxide layer plays a negligible role in most photonic-crystal cavities, and it has generally been ignored in previous works, but the strongly localized

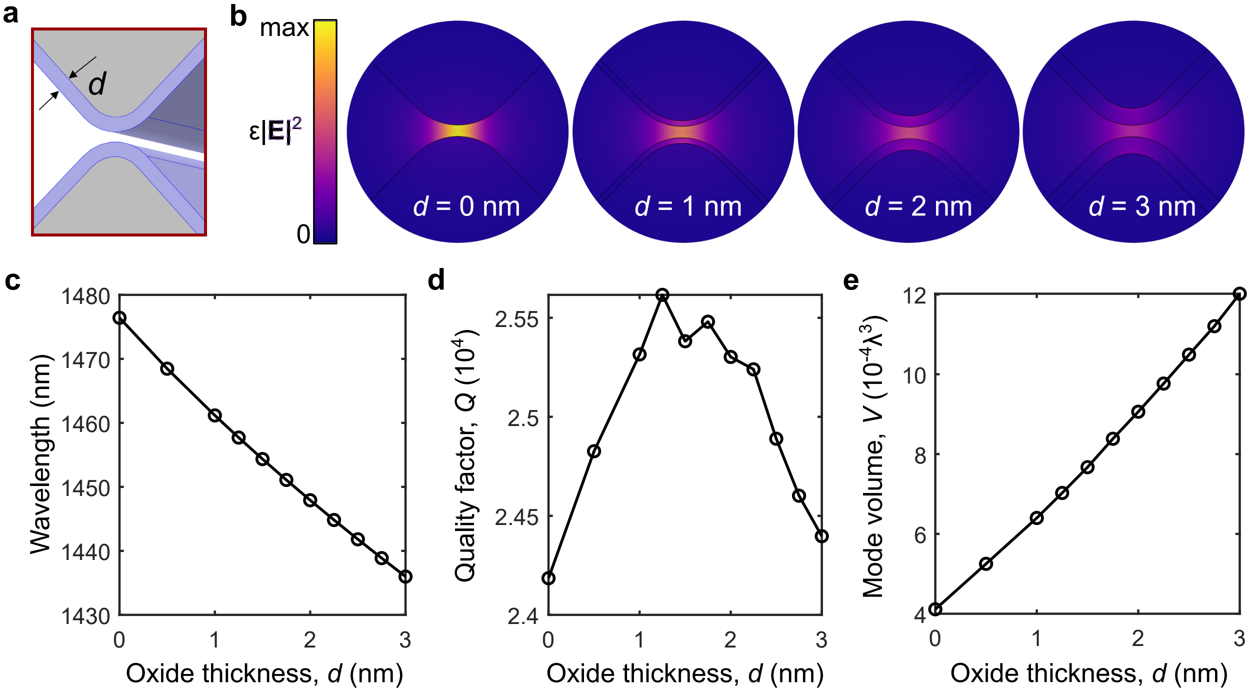

Supplementary Figure S15. **The role of the native oxide on the modal properties of air bowtie nanocavities.** **a**, Schematic of the conformal native silicon oxide film (blue) in the bowtie region. **b**, Electric energy density in the air bowtie region of the central unit cell of a cavity with the same geometric parameters as that simulated for Fig. 4 in the main text and varying oxide thickness, d. **c, d, e** The simulated cavities wavelength, quality factor, and mode volume as a function of d.

fields of bowtie cavities make their modal properties very sensitive to the thickness of the oxide layer. We illustrate the strong influence of the oxide layer via simulations of the fabricated bowtie cavity shown in Fig. 4 in the main text with varying native oxide thickness, d. Figure S15a shows the geometry of the simulated bowties with a fixed air gap of $g = 2$ nm. The effect of the added oxide layer is to reduce the index contrast of the first interface from $\Delta n = n_{\mathrm{Si}}$ - n_{air} to $\Delta n = n_{\mathrm{oxide}}$ - n_{air}, where the different indices are given by $n_{\mathrm{Si}} = 3.48$, $n_{\mathrm{oxide}} = 1.45$ and $n_{\mathrm{air}} = 1$. Adding the native oxide layer decreases the field intensity in the bowtie, as evidenced by the energy densities shown in Fig. S15b. The impact of such field redistribution is negligible on the cavity quality factor, Q. Still, it produces a pronounced blue-shift of approximately 14 nm per nm of oxide (Fig. S15c) and, more importantly, leads to a more than two-fold increase in the effective mode volume evaluated at the bowtie center, V, between a realistic cavity with 2 nm of native oxide and the idealized cavity with no oxide

(Fig. S15e). We note that the oxide layer is neither included in the collapsed surfaces nor the top and bottom surfaces of the slab to limit the number of required mesh elements in the numerical model. These simulations show that previously reported values of mode volumes of bowtie cavities, which so far ignored the surface oxide, must be interpreted with great care. This adds to the already known [13] numerical issues associated with lightning-rod surface effects, which are particularly important for bowtie cavities and can give rise to numerical artifacts if not handled correctly.

S3.5. The role of disorder

The prominent role of the native oxide reported in the previous section indicates, more generally, that perturbations to the boundaries of the bowties have a strong influence on the cavity parameters. This stems from the tight field confinement within the bowtie and the intense fields at their boundaries (see Fig. S15b). Therefore, the presence of sidewall and line-edge roughness (see Fig. S31 in Section S5.2), bowtie-width fluctuations (see Fig. S21 in Section S4.3), and imperfect hole positioning could potentially play a very significant role in bowtie cavities. In Fig. 3 in the main text, we observe that the measured quality factors, Q, drop by approximately a factor of 4 relative to the designed values, Q_{int}, which we attribute to sidewall roughness at the bowtie tips and at the collapsed interface. This drop occurs even if the cavities are designed to exhibit rather small values of Q_{int}, which are well below the experimental values previously demonstrated ($Q \sim 7 \cdot 10^5$) with our fabrication process [17, 18]. In this section, we present numerical simulations aiming to identify the origin of the observed experimental values for Q.

Modeling the effect of disorder in nanophotonic devices is a major outstanding challenge. Firstly, this is because such models require detailed knowledge of the statistical properties (amplitude and disorder correlation) of all kinds of disorder (sidewall roughness, surface roughness, line-edge roughness, sidewall-angle fluctuations, shape disorder, positional disorder, chemical contamination from etching, etc.). Secondly, it requires numerical schemes that can handle the models with realistic computational resources. Finally, the numerical challenges are increased manyfold by the fact that disorder is a statistical phenomenon, so many realizations must be considered in order to build up ensemble information. As a con-

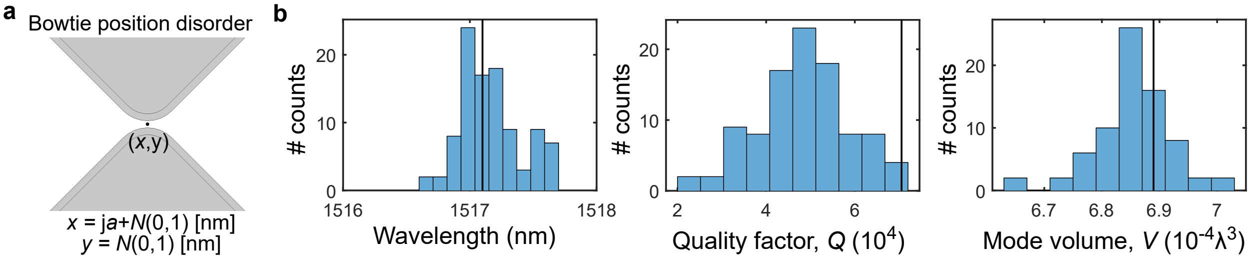

Supplementary Figure S16. The effect of structural disorder on the modal properties of air bowtie nanocavities. a, Bowtie-tip geometry, indicating the presence of positional disorder, with disorder considered as a departure from the nominal centroids by a quantity determined from a normal distribution, $\mathcal{N}(0,1)$. **b** Histogram of the resonant wavelengths, Q-factors, and mode volumes, V, obtained from 100 cavity realizations. The vertical lines in the histograms indicate the values obtained for the unperturbed case.

sequence of these factors, the typical approach in the literature is either to use perturbation theories [19] or to assume that all kinds of disorder can be projected onto a single kind [20].

While we have directly included line-edge roughness in a recent publication [18], the finite-element quasi-normal-mode calculations performed here are already computationally heavy in the absence of disorder. This is due to the number of mesh elements required to converge the figures of merit of cavities (see Section 3.3) with critical dimensions at the nanometer or subnanometer scale. Modeling surface roughness at a scale below a nanometer would require dramatically finer meshes, making full-scale simulations of even a single cavity realization an intractable problem with our resources. In addition, our attempts at employing non-Hermitian first-order perturbation theory [19] to explain our experimental observations fail since they are unable to capture symmetry-breaking in our cavities, which is inherent to the actual roughness, and the main reason behind the shift of the overall distribution of measured Qs towards lower values than the designed ones [21].

Based on the aforementioned considerations, we project the various observed kinds of disorder into the only type of disorder that is numerically tractable while capturing the full breaking of symmetries that disorder imposes, namely fluctuations in the centroid of the bowties. This reduced disorder model has been recurrently used in the literature on photonic-crystal structures [20, 22, 23]. For the particular case we explore here, full-scale simulations are at reach despite the added computational cost of increasing the domain to

one-half of the full nanobeam (the symmetry relative to the mid-plane is preserved since the sidewalls are still considered to be vertical), instead of one-eighth. We simulate the fundamental mode of 100 cavity geometries that include normally distributed and uncorrelated xy-position fluctuations, $\mathcal{N}(0, 1)$, of the bowtie centroids (Fig S16a). Figure S16b shows the histogram of their calculated resonance wavelength, Q-factor, and mode volume. While wavelengths and mode volumes are distributed around the unperturbed cavity values, we observe a 37% reduction of the mean Q relative to the unperturbed Q. Although our cavities were designed to use a fixed integer shot pitch, which fits the 1 nm electron-beam-lithography exposure grid and therefore minimizes centroid disorder in the lithography, fluctuations during development and etching will in general break the lateral mirror symmetries. It is precisely this breaking of the symmetry that degrades Q, and therefore we attribute the drop in Q to a combination of positional disorder and the symmetry-breaking effect of sidewall roughness, which is only partially and approximately captured by our model, which assumes that all kinds of disorder can be projected onto positional disorder only.

S4. SELF-ASSEMBLY OF AIR-BOWTIE CAVITIES

Our fabrication process uses conventional steps for lithography, dry etching, and selective underetching. In this section, we discuss details of these processes and their relevance towards achieving self-assembled air-bowtie of few to sub-nanometer gaps.

S4.1. Silicon etch and sidewall verticality

The etching sequence utilizes a fluorocarbon-free and low-power switched reactive ion-etching process. It employs O_2 for sidewall passivation and SF_6 for silicon etching [13, 24]. To achieve high-resolution features with electron-beam lithography [13, 25], thin resists (soft masks) are necessary. However, due to their limited selectivity to plasma etching, thin resists result in roughness and often also non-vertical sidewalls. Additionally, small features and narrow trenches are limited by mask retraction and lateral mask etching when using thin resists. In contrast, hard masks exhibit high resistance to plasma etching, allowing for

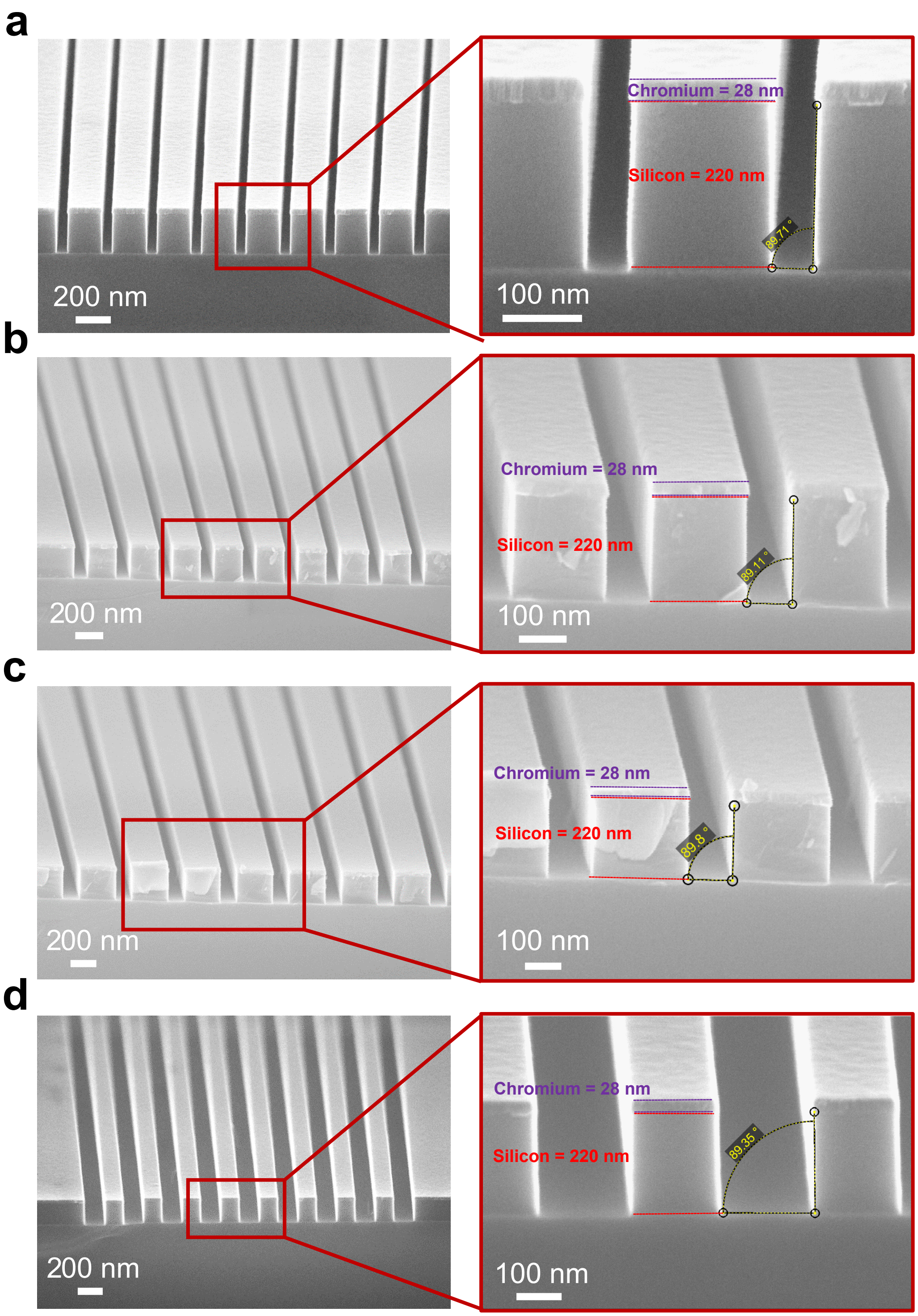

Supplementary Figure S17. **Etching profile and sidewall angle of trenches in a 220 nm silicon device layer.** **a**, 50 nm wide trenches. **b**, 90 nm wide trenches. **c**, 120 nm wide trenches, **d**, 200 nm wide trenches.

shorter cycle times with minimal mask retraction or lateral mask etching [26]. Therefore,

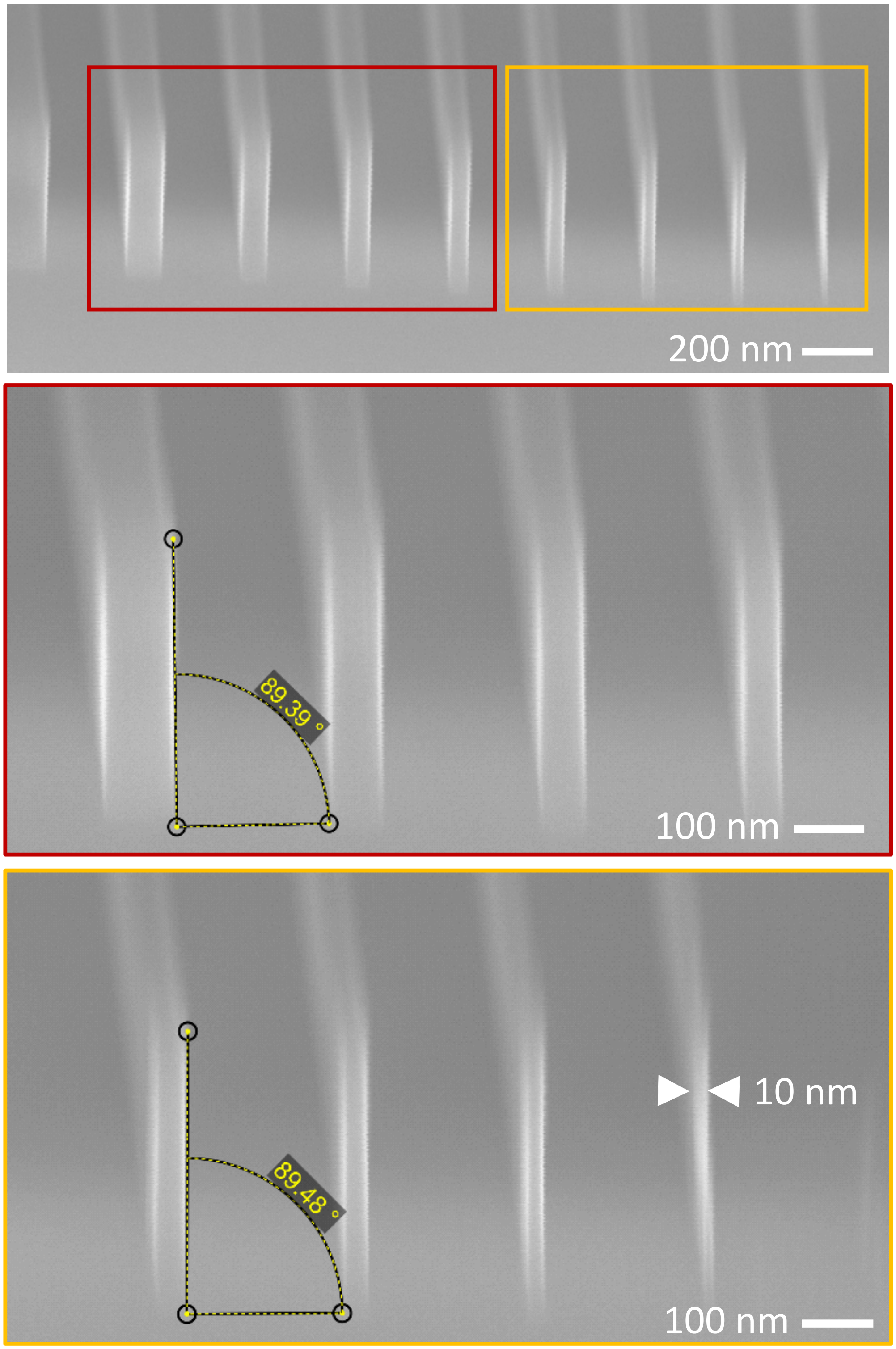

Supplementary Figure S18. Etching profile and sidewall angle of lines with different widths in a 220 nm silicon device layer.

our etching process utilizes a 12 nm poly-silicon and 30 nm poly-chromium hard mask to

etch into our 220 nm silicon device layer (see Methods and Refs. [17, 27] for details on the hard-mask etching process). Poly-silicon is an intermediate mask to transfer the pattern from a 50 nm chemically semi-amplified resist (CSAR) into chromium with high selectivity and fidelity. The chromium etching involves a two-step process comprising an oxygen-rich and fluorine-rich step, accomplished with 15 cycles [27, 28]. Using chromium as a hard mask enables the etching of silicon with shorter cycles, resulting in smoother sidewalls and reduced scallops, which are crucial for high-Q nanocavities. We adopt 25 cycles to etch the 220 nm silicon device layer. At the end of the process, chromium is removed from the silicon device layer through a chromium etch step involving 15 cycles. The SEM images in Figure S17 depict the etch profiles of four different etch tests, featuring trenches of 50 nm, 90 nm, 120 nm, and 200 nm widths in the silicon device layer, all etched using the hard-mask process. The etching quality is excellent across the studied range of trench widths. Notably this includes the 50 nm trenches, which we use in our experiments. Eventually, for trenches much smaller than 50 nm, the sidewalls develop a significant slope, and the etching quality is reduced, but a main benefit of our surface-force-assisted self-assembly is that we never need to make lithographic gaps below 50 nm, even to reach subnanometer gaps in the self-assembled device. The etch profiles exhibit sidewall angles of approximately 89° to 90°, irrespective of the size, which is important, for the self-assembly process in creating sub-nanometer scale voids. This sidewall angle is significant as it determines the strength of surface forces and ensures self-parallelism of the sidewalls during self-assembly (see the STEM discussion in Section S4.4). Additionally, Figure S18 presents narrow ridges in a 220 nm silicon device layer using the same hard-mask process, demonstrating the achievement of solid features, such as walls, as narrow as 10 nm with straight and nearly vertical sidewalls (89° to 90°). The minimum width for the etched ridges is considerably smaller than that for the etched trenches. This is firstly because it is much harder to do proximity-effect correction for small gaps than for small ridges as it is always possible to add more electron dose to a ridge to bring the exposure above the clearance dose, but it is not always possible to remove enough dose from a gap to bring it below clearance (if the surrounding features require so high a dose to be cleared that the proximity dose in the gap is nearly at clearance). Secondly, micro-loading effects during dry etching are also much more severe for small gaps than for small ridges (because the ridges can be surrounded by large features for which the micro-loading effects can be made small).

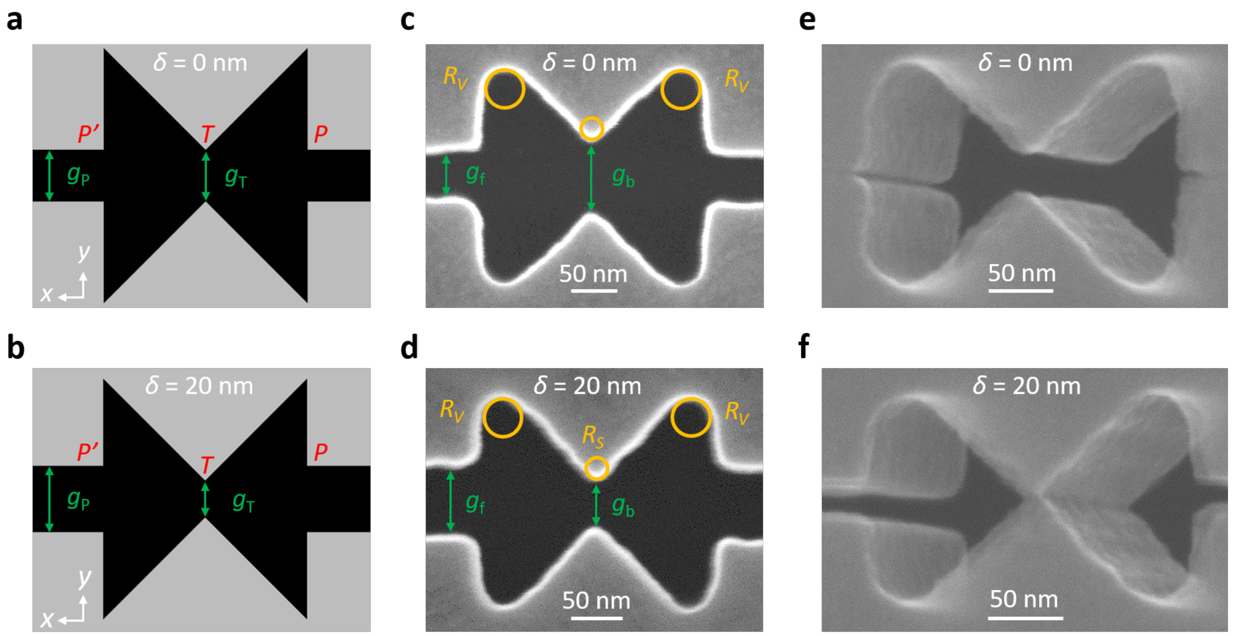

Supplementary Figure S19. **Deterministic fabrication of few-nanometer bowtie widths. a, b**, Schematic of the electron-beam lithography mask for a bowtie unit cell. The exposed region is colored black, and the grey region corresponds to silicon features after exposure and reactive ion-etching. The bowtie unit cell comprises two unconnected halves with a nanobeam gap, g_P, and a bowtie gap, g_T. The relative distance between point T and the line PP' is given by the offset, δ, which is 0 nm for (**a**) and 20 nm for (**b**). **c, d**, Top-view SEM images of bowtie unit cells fabricated using (**c**) $\delta = 0$ nm and (**d**) $\delta = 20$ nm, after lithography and plasma etching but before self-assembly. The finite solid and void radius of curvature, R_S and R_V respectively, are indicated. **e, f**, Tilted-view (20°) SEM images of a bowtie unit cell with (**e**) $\delta = 0$ nm and (**f**) $\delta = 20$ nm after the self-assembly process.

S4.2. Deterministic fabrication and size control of air bowties

In the cavity design shown in Fig. S10, all feature sizes except for the few- or sub-nanometer air-bowtie widths can be fabricated using conventional lithography and etching. Figures S19a and b show schematics of the lithography mask used to self-assemble a single bowtie unit cell with a controlled air-bowtie width. Relative to the final self-assembled geometry, the mask structure is composed of two unconnected regions that are separated by a gap of width g_P along the flat parallel boundaries and by a gap of width g_T between the two bowtie tips.

We define the offset, δ, as the relative distance between one of the bowtie tips (T) and the line defined by the flat edges (PP'), i.e., $\delta = (g_\mathrm{T} - g_\mathrm{P})/2$. For example, $\delta = 0$ defines a bowtie unit cell where T, P and P' are co-linear, and g_T and g_P are equal. In contrast, $\delta = 20$ nm defines a bowtie unit cell where the relative vertical distance between PP' and T is 20 nm, as shown in Figs. S19a and b. The reason for having the tips closer to the central axis than the flat edges (PP'), i.e., $g_\mathrm{P} > g_\mathrm{T}$, is that the fabrication process rounds all sharp features to finite radii of curvature. We have estimated approximately $R_\mathrm{S} = 10$ nm for silicon features and $R_\mathrm{V} = 20$ nm for void features for our fabrication process [13]. Therefore, the top and bottom tips of the bowties after fabrication are effectively retracted from each other, as shown in the top-view SEM images of Figs. S19c and d, which are aquired before underetching. In addition to changing the offset, δ, we adjust the bowtie dimensions to preserve its 90° angle, which minimizes shot-filling and fracturing issues during electron-beam lithography. Since the surface forces mainly originate from the parallel surfaces of the nanobeams, the offset is varied by keeping g_f fixed at 50 nm (well within the borders of our fabrication process, c.f. Section S4.1) while changing g_b. Examples of the resulting air bowties are shown in the tilted-view SEM images of Figs. S19e and f for $\delta = 0$ nm and $\delta = 20$ nm. The former produces an air bowtie at the unit cell center while the tips are in contact with the latter, which showcases another type of application of the proposed self-assembly in which locally protruding regions are used as stoppers, allowing the formation of few-nanometer-wide slot waveguides. In the next section, we demonstrate the deterministic fabrication of few-nanometer gaps by varying the offset between the two extreme cases discussed here.

As a mask parameter, δ is subject to the restrictions of the lithography system. In our case, we use an exposure pitch of 1 nm. This implies that the tip-to-tip distance in our mask is changed in steps of 2 nm since we preserve the mirror symmetry, which is good design practice for nanomechanical devices. Therefore, in the absence of fluctuations, the most generic gap-to-offset correspondence, $g(\delta)$, we can expect for our implementation of the proposed self-assembly is

$$g(\delta) = 2\delta + C, \quad \delta \in Z \tag{S6}$$

It is important to stress that this mask limitation of 2 nm steps is not transferred directly to the fabricated device in a more generic setting because the electron dose could be varied

in very fine steps. The gaps can therefore be made arbitrarily small – although not smaller than the surface roughness as discussed in Section S1. In the particular approach we employ, i.e., the use of δ as the only design parameter controlling the gap in the final device, and assuming no fluctuations on the edge definition, the achieved gaps are single-digit nanometer and can achieve the sub-nanometer regime depending on the exact value of C in Eq S6. The value of C depends on several process-dependent parameters. Two of them are the uniform enlargement, Δe, of all exposed features (see Section S1) and the solid radius of curvature, R_{S}. While we observe the latter to remain stable from sample to sample, Δe varies, which we attribute to a number of effects:

- **Proximity effects.** Even if we are using advanced proximity effect correction (PEC) on the lithographic mask using Beamfox Proximity, we observe that the size of specific features, e.g., the gap and triangle in the nanobeam, depends on the size of other neighbouring exposed areas, e.g., the trenches that define the nanobeam. The reason is that no proximity-effect correction algorithm has so far been able to rigorously capture and correct for the short-range proximity effects. While these effects are irrelevant in many cases, they are certainly important when fabricating devices whose ultimate (self-assembled) critical dimension drops below one nanometer. With this in mind, an inspection of the lithography mask reveals the origin of the differences between the different devices: The cavities for resonant scattering measurements consist only of a nanobeam cavity and folded guided cantilevers. On the other hand, the waveguide-coupled cavities have a much larger exposed region around them, consisting of a tapered section, waveguide-to-waveguide trenched couplers and long waveguide segments, including grating couplers. The extra dose which comes from exposing these components makes Δe larger for the Sample used in the results shown in Fig. 4 in the main text and Section S5.2 here.

- **Fabrication-process drift and fluctuations.** We observe Δe to drift and fluctuate over time as the process itself drifts. This is unsurprising given that we fabricate our devices in a shared university cleanroom with lithography and etching tools that run many different processes. Generally, we observe Δe to grow when either the lithography or dry-etching parameters depart from optimal conditions, which occurred when fabricating the chip used for the waveguide-coupled cavities of Fig. 4 in the main

text. An indication of that air-region growth is given by the resonant wavelengths of the waveguide-coupled cavities, which are considerably blue-shifted relative to the standalone resonant-scattering ones even if the bowtie widths are similar.

In principle, the value of Δe would be conformal for a lithography mask defined within the limits of the design rules (as discussed in Section S4.2), and in that case the gap would be independent of Δe. Our mask has sharp corners and there are additional effects of mask erosion and proximity effects at the tips of the bowties, which make Δe vary locally. Under simplifying assumptions on how the bowtie is formed, the enlargement at the bowties tips can be cast as a function of the enlargement Δe elsewhere. The resulting bowtie width, g, after self-assembly may be written as

$$g = 2((\Delta e + R_\mathrm{s})(\sqrt{2} - 1) - \delta) \tag{S7}$$

which determines the constant C in Eq. S6 and correlates it to measurable process parameters. For the cavity devices in this work, Δe is measured to be ∼3 nm (resonant-scattering measurements) and ∼13 nm (waveguide-coupled cavities), which we evaluate as half the change in the width of the gap from the lithography mask to the pre-underetched sample (half the change from g_P to g_f, see Fig. S19). If these values are inserted into Eq. S7, we find that the offsets, δ_*, for which the slot has opened are 6 nm and 10 nm, respectively. While this simplified model fails to accurately predict the actual δ_*, respectively 11 nm and 13 nm, it does predict that for larger Δe we expect the same gap g for larger values of δ. The importance of the final gap width on the batch-specific parameters is why δ is spanned over a wide range of values in the samples we explore.

S4.3. Scanning electron microscope characterization of offset-to-width correspondence

We fabricate nanobeam cavities by varying δ from 0 to 20 nm in steps of 1 nm and acquire SEM images on large bowtie subsets to characterize the underlying relationship between the offset δ and the bowtie width, g, after self-assembly. Figure S20 shows a representative high-resolution tilted-view SEM image of a bowtie unit cell as δ is changed from 1 nm (top-left) to 20 nm (bottom-right). First, the bowtie width monotonically narrows until a given offset δ_*, where the bowtie tips touch, leading to no void formation at the center. Due to the

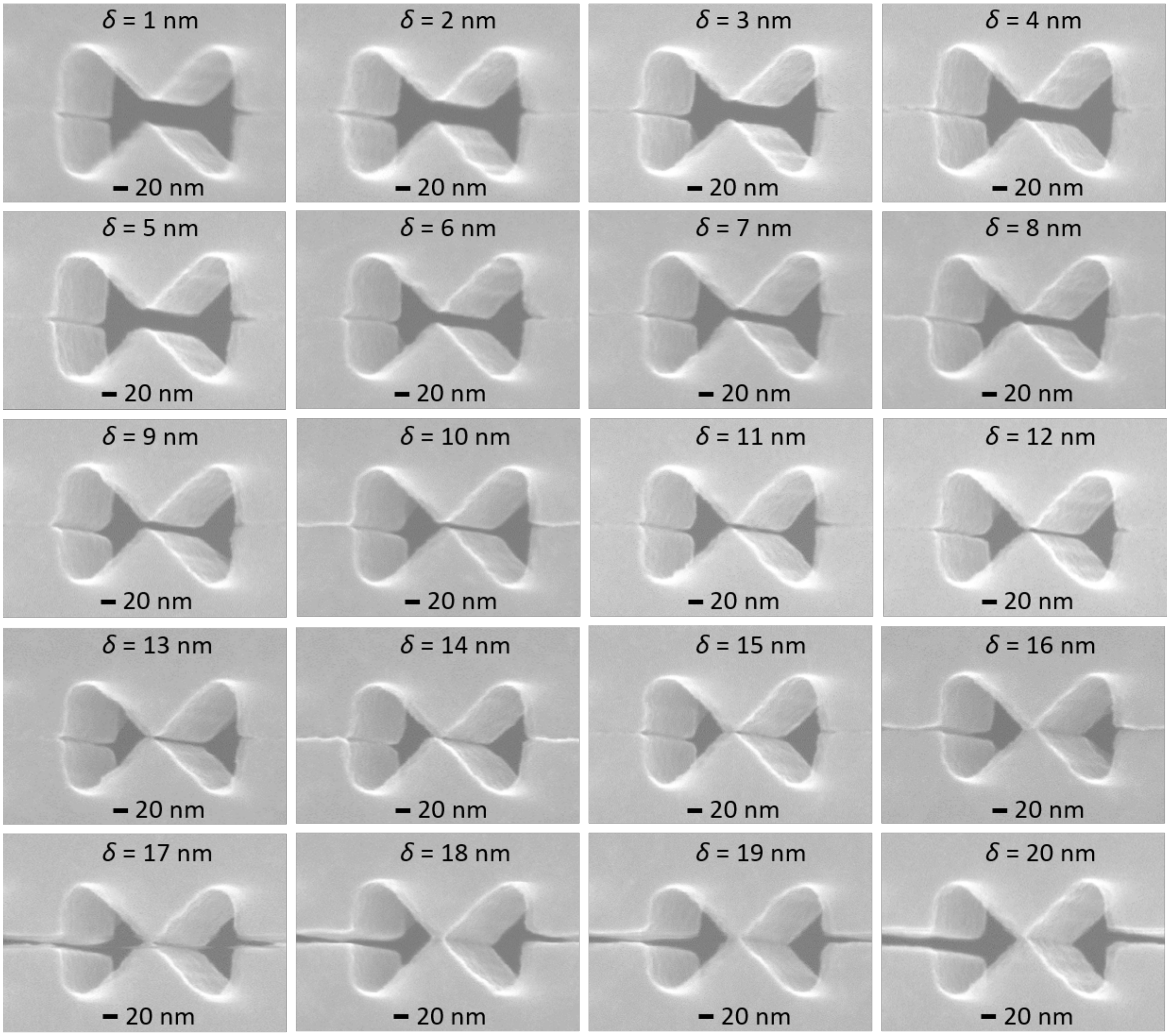

Supplementary Figure S20. **Characterization by SEM of few-nanometer bowtie widths.** Tilted-view (20°) SEM images of an array of self-assembled bowties with the offset, δ, varying from 1 nm (top-left) to 20 nm (bottom-right).

well-known systematic errors in SEM at the few-nanometer scale as well as bowtie-to-bowtie variations, the exact value of δ_* cannot be measured precisely, e.g., it is between 13 nm and 15 nm for the set shown in Fig. S20. As δ increases further, the bowtie tips start protruding towards the centre from the parallel surfaces and act as stoppers in the directed collapse, generating a slot whose width, denoted as g for simplicity, grows monotonically for even larger offsets. Note that, while all the structures in Fig. S20 are fabricated and self-assembled in a single fabrication run, other fabrication runs lead to a slightly different offset-to-width

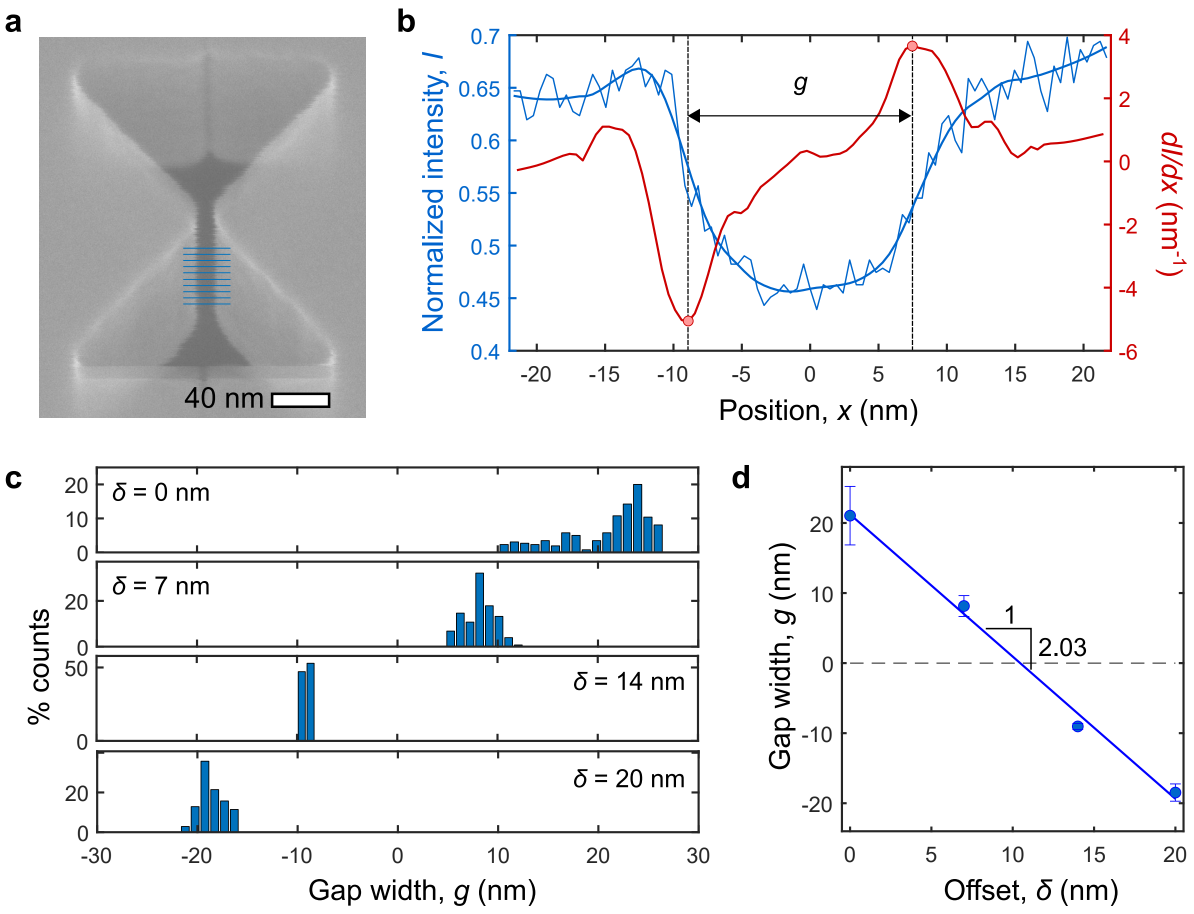

Supplementary Figure S21. **Extracting the relation between the mask offset, δ, and the fabricated bowtie width.** **a**, Tilted-view (20°) SEM image of a self-assembled air bowtie for $\delta = 10$ nm. The ten horizontal lines indicate cuts along which the gap, g, is extracted via image analysis. **b**, Normalized intensity along the first cut shown in **a** and its derivative. The most prominent maxima and minima in the latter are used to extract the gap. **c** Histograms of the extracted gaps for offsets 0, 7, 14 and 20, from which the average gap is extracted and shown in **d**, which includes a linear fit (solid blue line).

correspondence, as is, for example, the case for the structures reported in Fig. 3 in the main text. In that particular sample, the offsets are limited to $\delta = \{0,7,8,9,10,11,12,13,14,20\}$ nm, which are chosen to ensure covering the regime of few-nanometer air-bowtie cavities while including two cases in which the structure exhibits wide bowties ($\delta = 0$ nm) and wide slots ($\delta = 20$ nm) to help find the precise offset-to-width relation without being limited by the SEM resolution or artifacts.

We image all the bowtie unit cells in the cavity region of several nanobeams fabricated

with offsets 0, 7, 14, and 20 and extract the gap width in 10 positions across the device layer for each image, as exemplified in Fig. S21a. This approach of using multiple cuts is taken to account for the combined effects of the granularity of the pixel-based imaging and the tilt of the bowtie axis, and to investigate the verticality of the gaps, which we measure to be near-perfect (see discussion in Section S4.1 for our explanation). At each position, g is extracted via edge detection using the maximum derivative points in a smoothed version of the SEM image intensity (Fig. S21b). Figure S21c depicts the histogram obtained for the four used offsets, while Fig. S21d depicts a linear fit to the average gaps that we use to estimate the effective widths at all other values of δ, notably the bowtie widths of 5.0 nm, 3.0 nm and 1.0 nm used for $\delta = 8$, 9 and 10 in Fig. 3b in the main text. We highlight that the extracted slope of 2.03 is in excellent agreement with the value of 2 one may expect from the mask geometry and that the curve intersects the horizontal axis approximately $\delta_* = 11$ nm. Even if the extracted offset-to-gap correspondence indicates a minimum bowtie width of 1 nm, the bowtie-width fluctuations for a fixed value of δ are larger than 1 nm, i.e., the average of the standard deviations of the different distributions is 1.8 nm, and nanobeam cavities fabricated with $\delta = \delta_* - 1$ exhibit sub-nanometer gaps.

S4.4. Scanning transmission electron microscope characterization of bowtie widths

To precisely characterize the bowtie width for values of δ around δ_* and the native oxide layer, we fabricate self-assembled cavities for high-resolution STEM imaging. Since STEM requires thin layers of material to transmit electrons for analysis, individual nanobeam cavities are cut out from the wafer chip using a focused ion beam (FIB) and transferred to STEM-compatible grids (see Methods for details on the lift-off process for STEM imaging). In the sample dedicated to STEM, we employ an anchoring system that assists in cutting and lifting off the cavities from the chip (see Fig. S22a). We select 5 devices from the sample with $\delta = 8$, 9, 10, 11, and 12 nm and transfer them to the TEM grids using a motorized micro-manipulator as shown in Fig. S22b. Figure S22c shows a top-view STEM image of a self-assembled nanobeam cavity and its central bowtie unit cell. Characteristic high-resolution STEM images of the central bowtie region for individual bowties belonging to cavities fabricated with δ from 8 to 12 nm are shown in Fig. S23. The non-monotonic

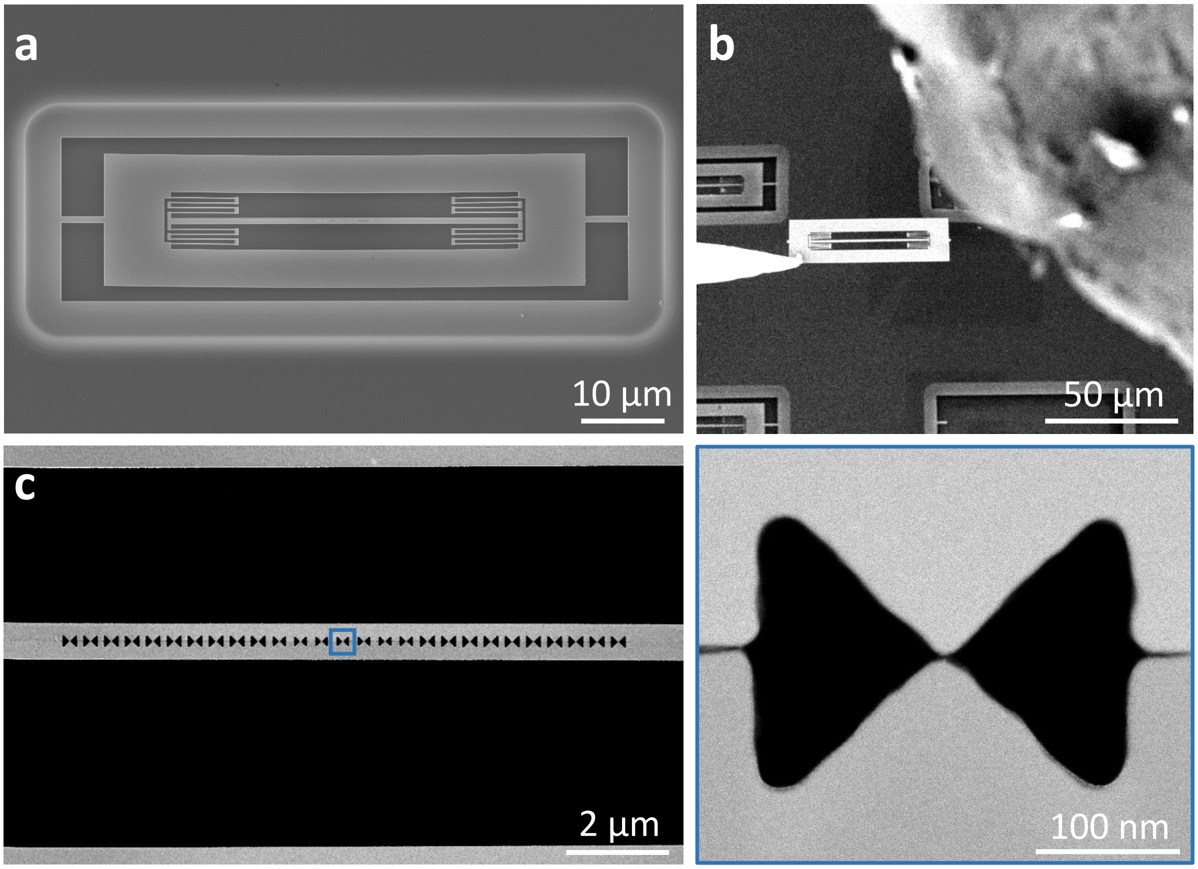

Supplementary Figure S22. High-resolution STEM imaging of self-assembled nanobeam cavities. a, Tilted-view (20°) SEM image of a self-assembled nanobeam cavity with the surrounding frame designed for improving the mechanical robustness of the structure to allow ion-beam milling and handling by micromanipulators. **b**, Transfer of a self-assembled nanobeam cavity to a STEM grid using a micromanipulator tip after removing the structure from the substrate using the focused ion-beam gun. **c**, Top-view STEM image of a self-assembled nanobeam cavity and a zoom-in into the central unit cell (blue box).

evolution of the bowtie width with δ indicates that extremes in the bowtie-width fluctuations for a fixed value of δ are at least larger than 1 nm, which is consistent with our SEM image analysis shown in Fig. S21). This indicates that nanobeam cavities fabricated with $\delta = \delta_*$ include bowties that exhibit sub-nanometer gaps and demonstrates in any case the ability of our method to fabricate cavities with atomic-scale gaps.

The signal intensity in STEM depends on composition, density, and thickness. The intensity decreases gradually when transitioning from the crystalline silicon to the amorphous

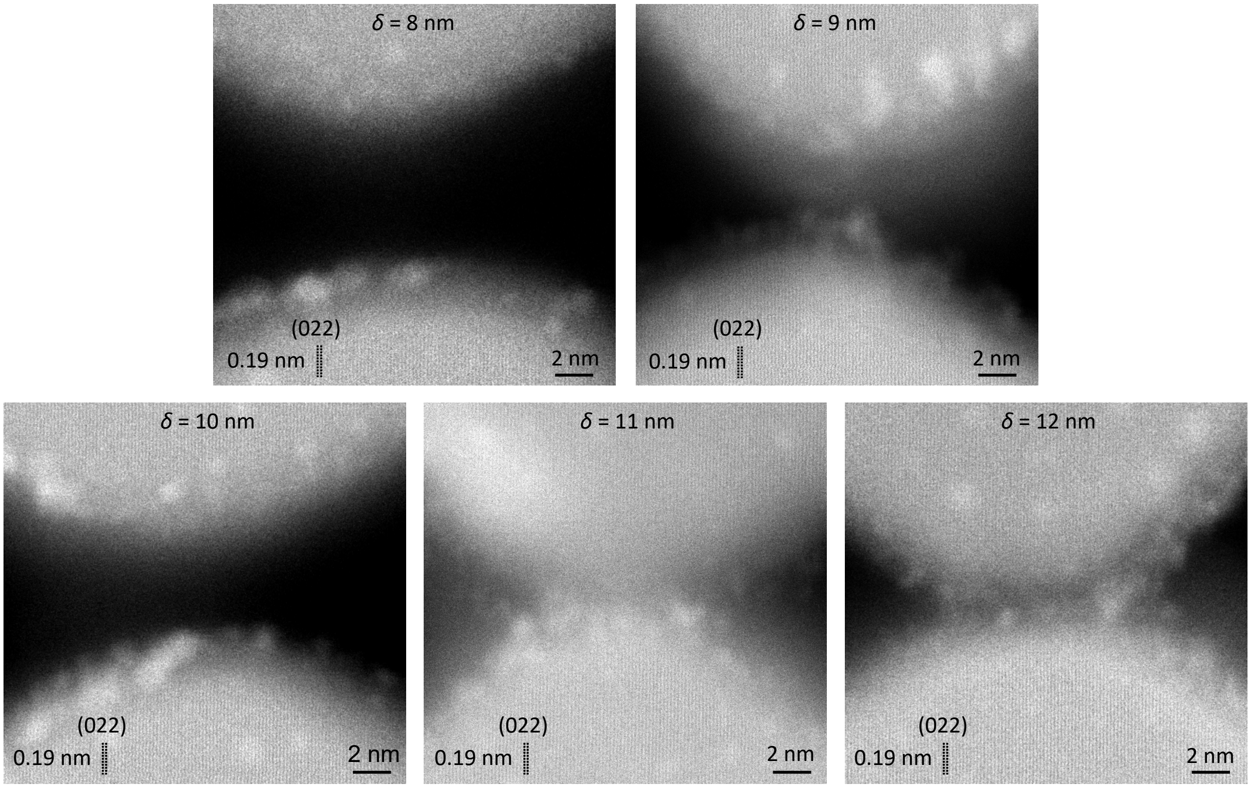

Supplementary Figure S23. **Characterization by STEM of atomic-scale bowtie widths.** Annular dark-field STEM images of the bowtie tips of the central unit cells in nanobeam cavities fabricated with δ from 8 nm to 12 nm. The (022) silicon crystal planes are observed and indicated in the images.

oxide and reducing to a background signal at the void region. Given the spatial resolution of approximately 0.1 nm of our STEM and the observations of the sidewall tilt and roughness discussed in the main text, we attribute the intensity drop to the bowtie geometry and composition, i.e., the thinner the probed thickness, the dimmer the intensity. We extract the thickness of the native oxide layer by considering its edges to be defined by the position where the crystalline lattice is no longer visible on the bottom half bowtie side and where the rate of change in intensity is maximum on the void side. Based on where the (022) lattice signal extends in the images, a 2-2.5 nm thick amorphous layer is measured at the edge of the structures, as confirmed by atomic composition analysis using electron energy-loss spectroscopy (EELS). The exact measurement is complicated due to the dependence of signal intensity on the thickness of the cross-section seen by the electron beam transmitted through the slightly tilted sidewall. As expected from the negative correlation between offset

and bowtie width, a pronounced air gap (void region) is observed for $\delta = 8$ nm. At offsets 11 and 12, the bowtie tips are in contact with the native oxide layers; therefore, the void region disappears. In between, the progressive change in the bowtie width is more complex than one would expect from the design rules described in Section S4.2 due to a combination of the roughness of the bowtie tips and the bowtie shape in the vertical direction.

S4.5. Self-assembled nanobeam bowtie cavities

The nanobeam bowtie cavities are fabricated as two halves, each suspended by two folded cantilevers with a total spring constant of 0.038 N/m. The two halves are separated by a gap, $g_\mathrm{f} = 50$ nm, such that the set $\{k,A,g_0\}$ lies deep within the parameter space leading to surface-force-assisted collapses (see Fig. S8) and therefore the two halves adhere after underetching. Figure S24 reproduces Fig. 2g in the main text and shows a characteristic device, which includes 22 μm of unpatterned half-beams on each side of the photonic-crystal bowtie cavity to increase the surface forces and aid the self-assembly, as well as the two pairs of folded cantilever springs. The cross-shaped etched trenches around the cavity structure are included for alignment purposes in the cross-polarized optical microscopy setup.

S5. OPTICAL SPECTROSCOPY OF AIR-BOWTIE NANOCAVITIES

S5.1. Far-field resonant scattering measurements

As detailed in the Methods section, we perform optical spectroscopy of the self-assembled nanocavities using far-field resonant-scattering measurements. The probed cavity mode has a polarization in the far-field that is mainly along the y-axis as indicated with the white arrow in the SEM image of Fig. S25a. We couple light into the cavity by exciting at normal incidence using linearly polarized light with a polarization of 45° relative to the cavity-mode polarization and collect light at 90° relative to the excitation polarization or -45° to the cavity polarization as shown in Fig. S25a. The excitation and collection with cross-polarization optimize the cavity-to-background contrast. The cavity resonances appear as Fano resonances (Fig. S25b and c) due to the interference between the high-Q in-plane cavity resonance and the low-Q out-of-plane resonance formed by the silicon membrane and the handle layer [13, 29]. Therefore, we fit our cavity spectra around the observed resonant

features using a Fano lineshape,

$$F(\omega) = A_0 + F_0 \frac{\left[q + 2\left(\omega - \omega_0\right)/\Gamma\right]^2}{1 + \left[2\left(\omega - \omega_0\right)/\Gamma\right]^2}, \tag{S8}$$

where A_0 and F_0 are constant scaling factors, ω is the frequency, ω_0 is the resonance frequency of the cavity mode, Γ its linewidth, and q quantifies the relative amplitudes between the cavity mode and the background modes. We note that q can have both positive and negative values and determines the lineshape asymmetry. We use a nonlinear regression to perform the fit and extract the quality factor from ω_0 and Γ, with the uncertainty, σ, computed by propagating the errors in ω_0 and Γ, which are obtained as the square root of the diagonal elements of the regression covariance matrix. In particular, we specify the uncertainty in Q as $\pm 2\sigma$ (95% confidence interval). The fitted lineshape, corresponding fit parameters, the coefficient of determination, R^2, and the extracted Q are shown in Figs. S26, S27, and S28 for the measured cavities, fabricated with δ values of 8 nm, 9 nm, and 10 nm, respectively. We note that most values of R^2 range between 0.95 and 1 for the fitted cavity spectra. The measured data set is summarized in Figs. S29a-c, which include SEM images

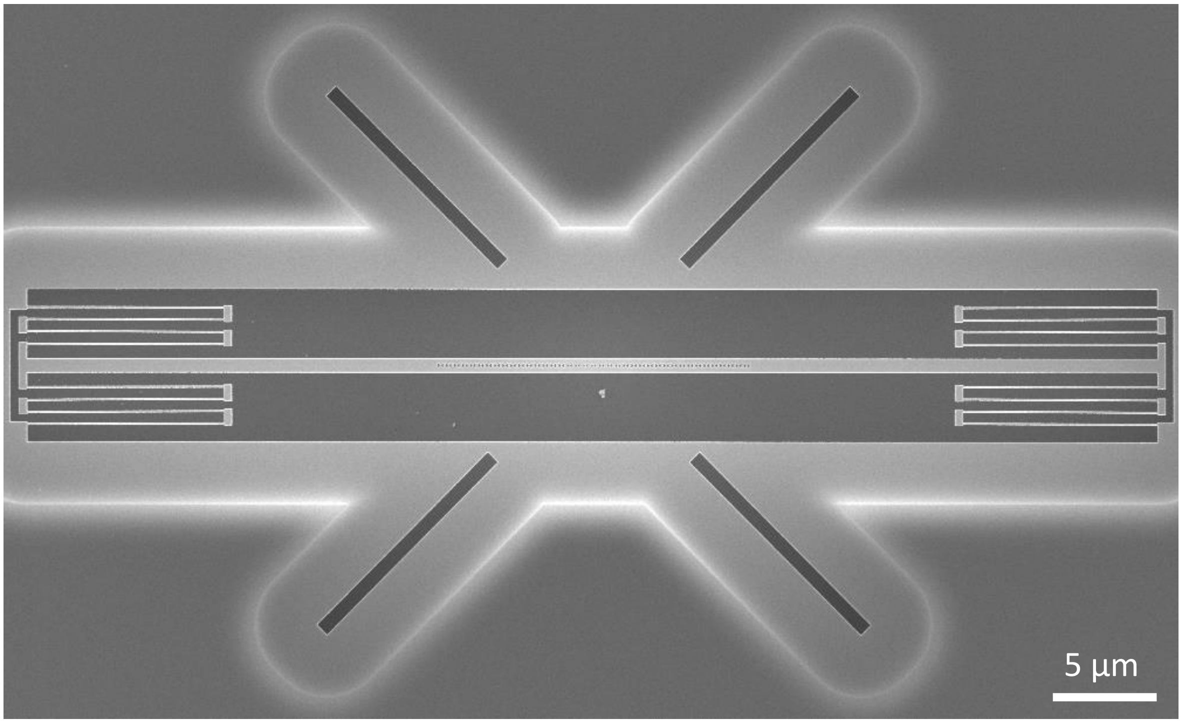

Supplementary Figure S24. Self-assembled nanobeam cavity with bowtie unit cells for δ = 10 nm. Top-view SEM image of the full device, including the spring suspension.

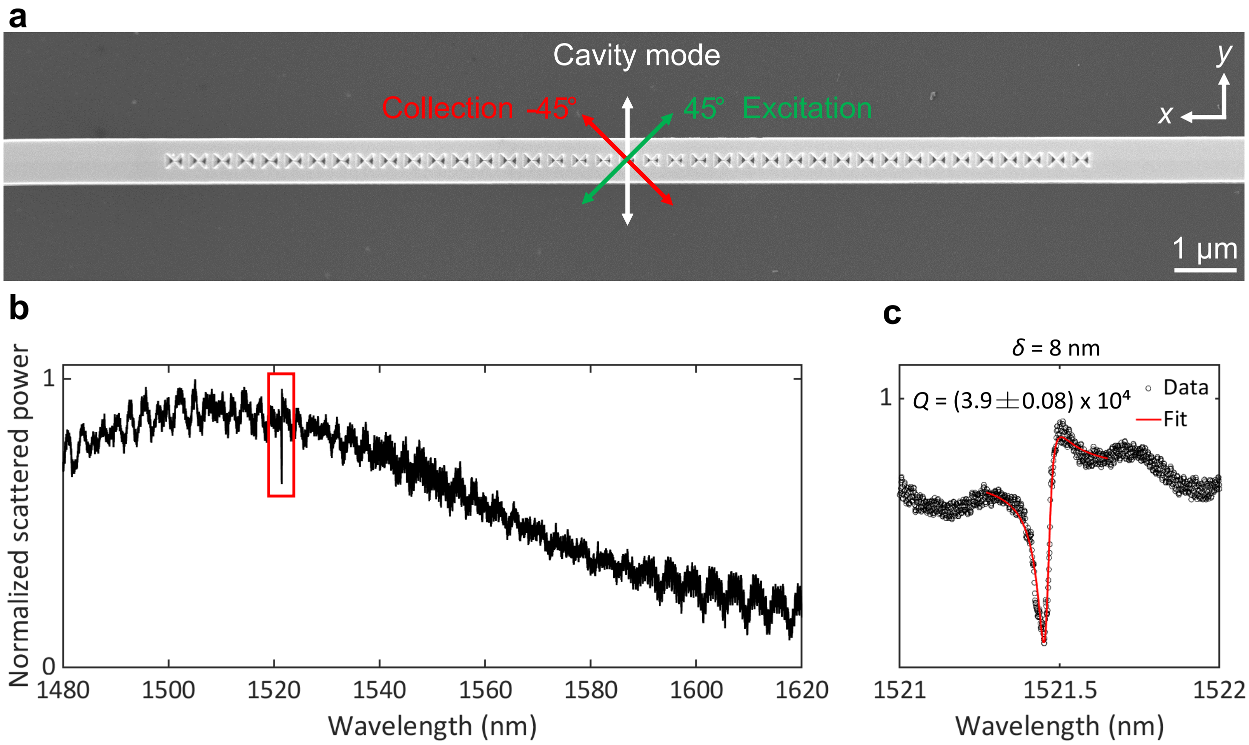

Supplementary Figure S25. **Cross-polarized resonant-scattering spectroscopy.** **a**, Tilted-view (20°) SEM image of a self-assembled nanobeam cavity showing the polarization of excitation (green arrow), detection (red arrow), and the cavity mode (white arrow). **b**, Normalized measured scattered power with excitation and collection at the cavity center. The red box highlights the cavity resonance, also shown in **c**. A fit to a Fano lineshape is overlayed in red, from which we extract the resonance wavelength and quality factor.

of the central bowtie of the measured cavities as insets. The resonance wavelengths exhibit a clear red-shift with increasing offset, i.e., decreasing bowtie width, and we measure quality factors between $(7.5 \pm 0.2) \times 10^3$ and $(4.2 \pm 0.01) \times 10^4$.

S5.2. In-line transmission measurements of self-assembled nanobeam cavities

We perform optical spectroscopy of waveguide-coupled self-assembled nanocavities using in-line transmission measurements, as detailed in the Methods section. Figure S30 shows a tilted-view SEM image of an entire waveguide-coupled self-assembled nanobeam cavity device, including the two circular grating couplers used for cross-polarized and spatially resolved excitation/collection. As discussed in the main text, the spectra obtained on such

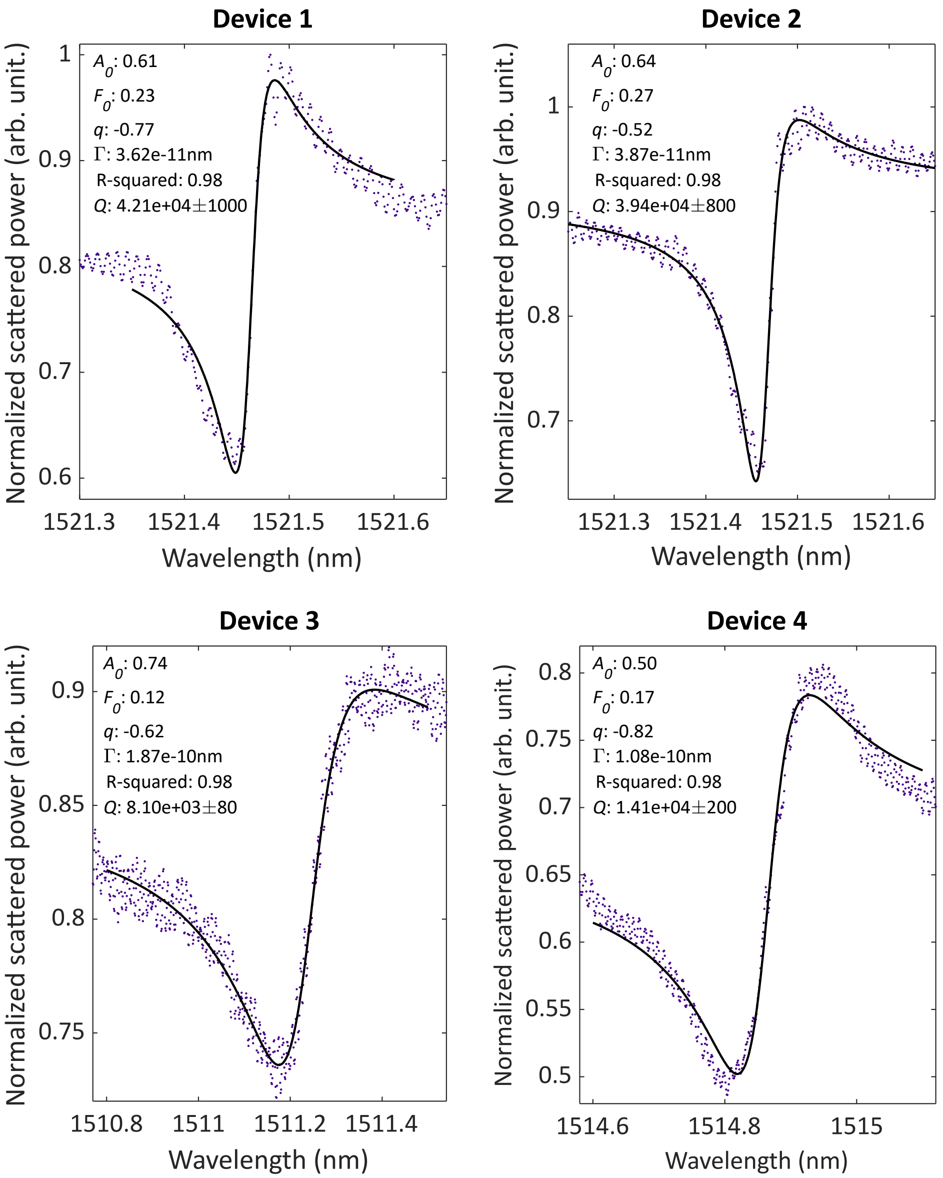

Supplementary Figure S26. **Fano lineshape and fitted parameters of cavity resonances for self-assembled cavities with $\delta = 8$.**

cavities are normalized to that measured on a self-assembled waveguide, a characteristic SEM image of which is shown in Fig. S31. The inset shows that the quality of the self-assembly is such that the interface is hardly visible.

Figure S32 shows measured transmission spectra (raw data) of a self-assembled nanobeam cavity, a self-assembled mirror waveguide, a self-assembled waveguide, and a conventional waveguide, all with equivalent length and integrated with tunnel-coupled waveguides interfacing to the external circuit, i.e., via waveguides and grating couplers. The cavity spectra

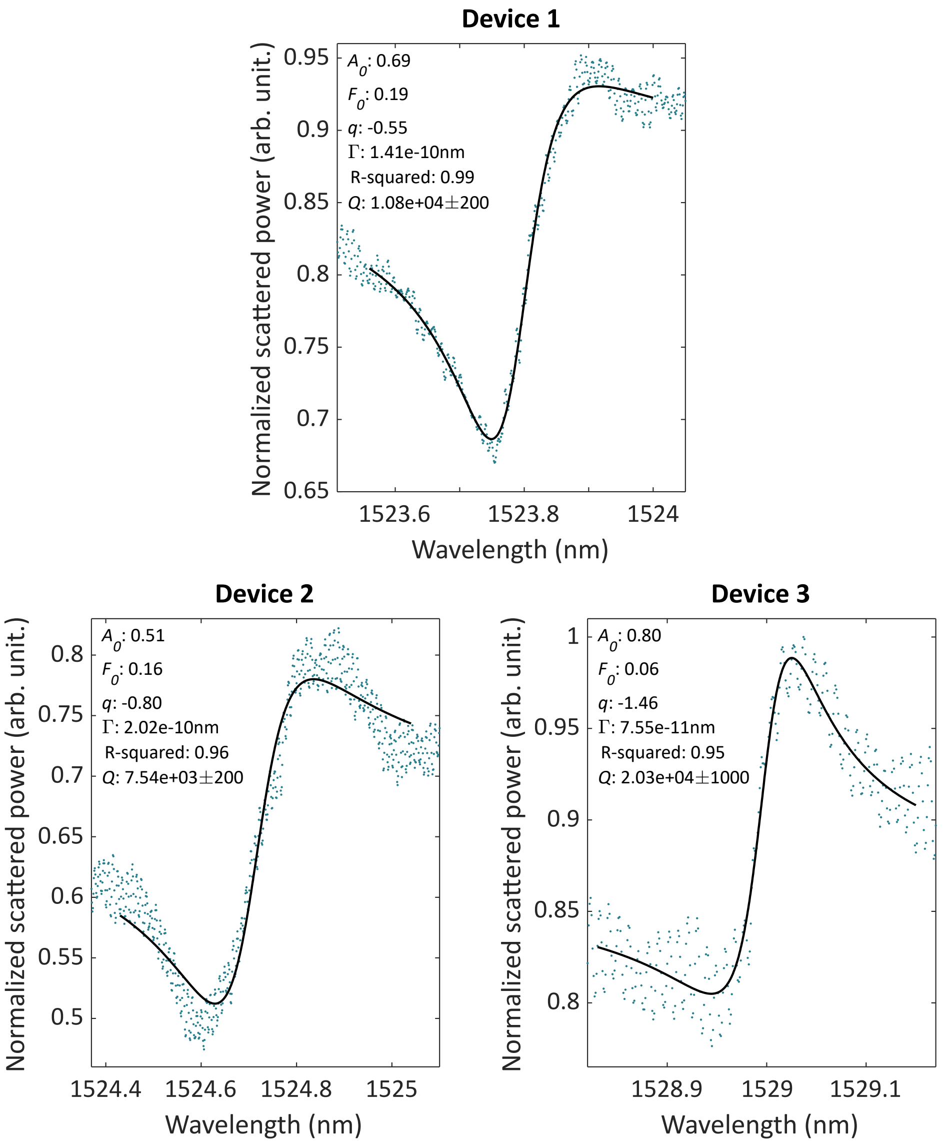

Supplementary Figure S27. **Fano lineshape and fitted parameters of cavity resonances for self-assembled cavities with $\delta = 9$.**

reported in the main text and in Figs. S33 and S34 are normalized to a smoothed version of the spectrum of the self-assembled waveguide (also shown in Fig. S32) to remove Fabry-Pérot fringes that result from strong reflections at the grating couplers. We observe a 25 % average drop in the waveguide transmission due to the self-assembly process, which stems from scattering losses due to the collapsed interface, although the possibility that the self-assembled and conventional waveguides bow differently (see for example Fig. 4c in the main text) cannot be ruled out. The bowing appears in our devices because they do not include stress-release structures. We also note that the transmission band observed for the

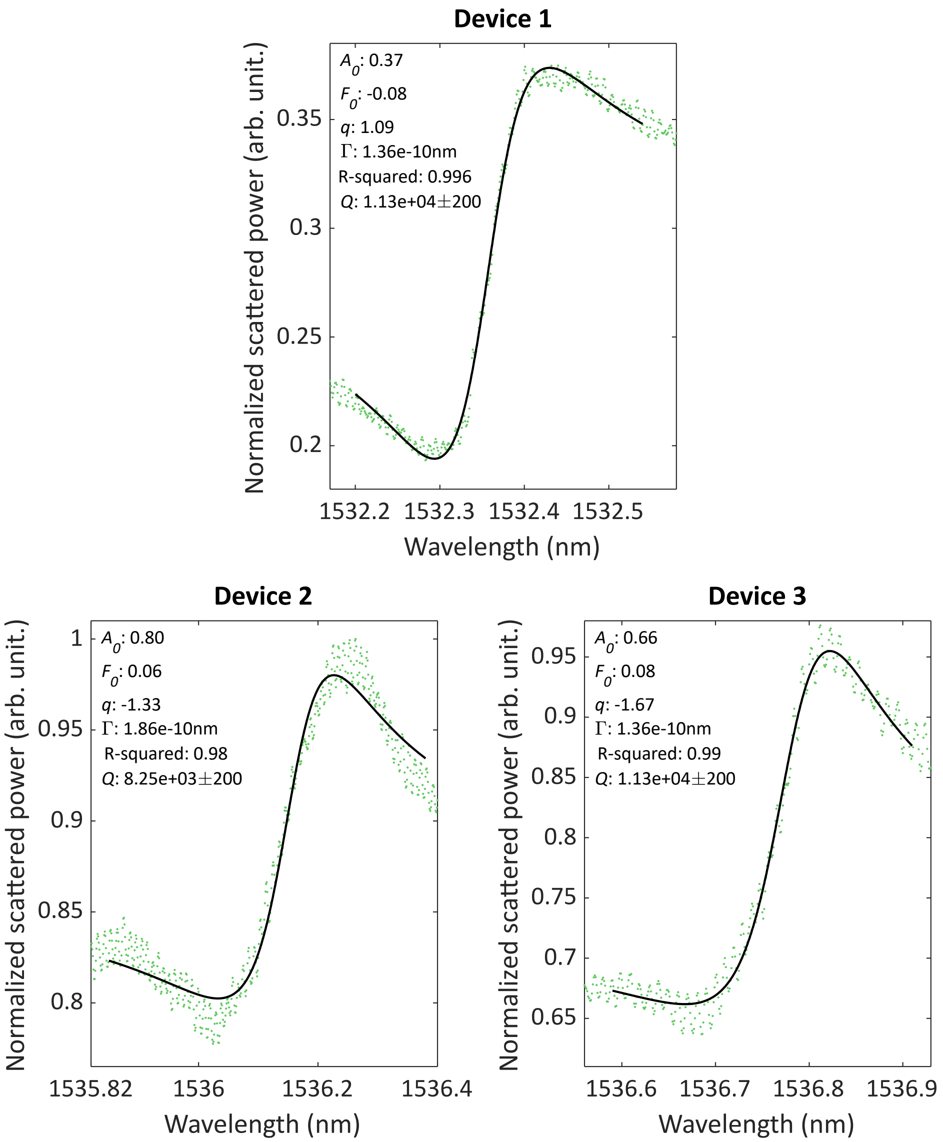

Supplementary Figure S28. **Fano lineshape and fitted parameters of cavity resonances for self-assembled cavities with $\delta = 10$.**

mirror waveguide at long wavelengths is not observed in the cavity spectrum, which results from the fact that the defect region forming the cavity modes at the short-wavelength band edge acts as a mirror at the higher-wavelength band edge (see Fig. S10b).

To demonstrate the compatibility and self-alignment of our self-assembly to surrounding circuitry, we fabricate five nominally identical circuits for the waveguide-coupled self-assembled nanocavity shown in Fig. 4 of the main text. Figure S33 shows the normalized transmission measurements for the set of copies. Lorentzian fits for the fundamental cavity

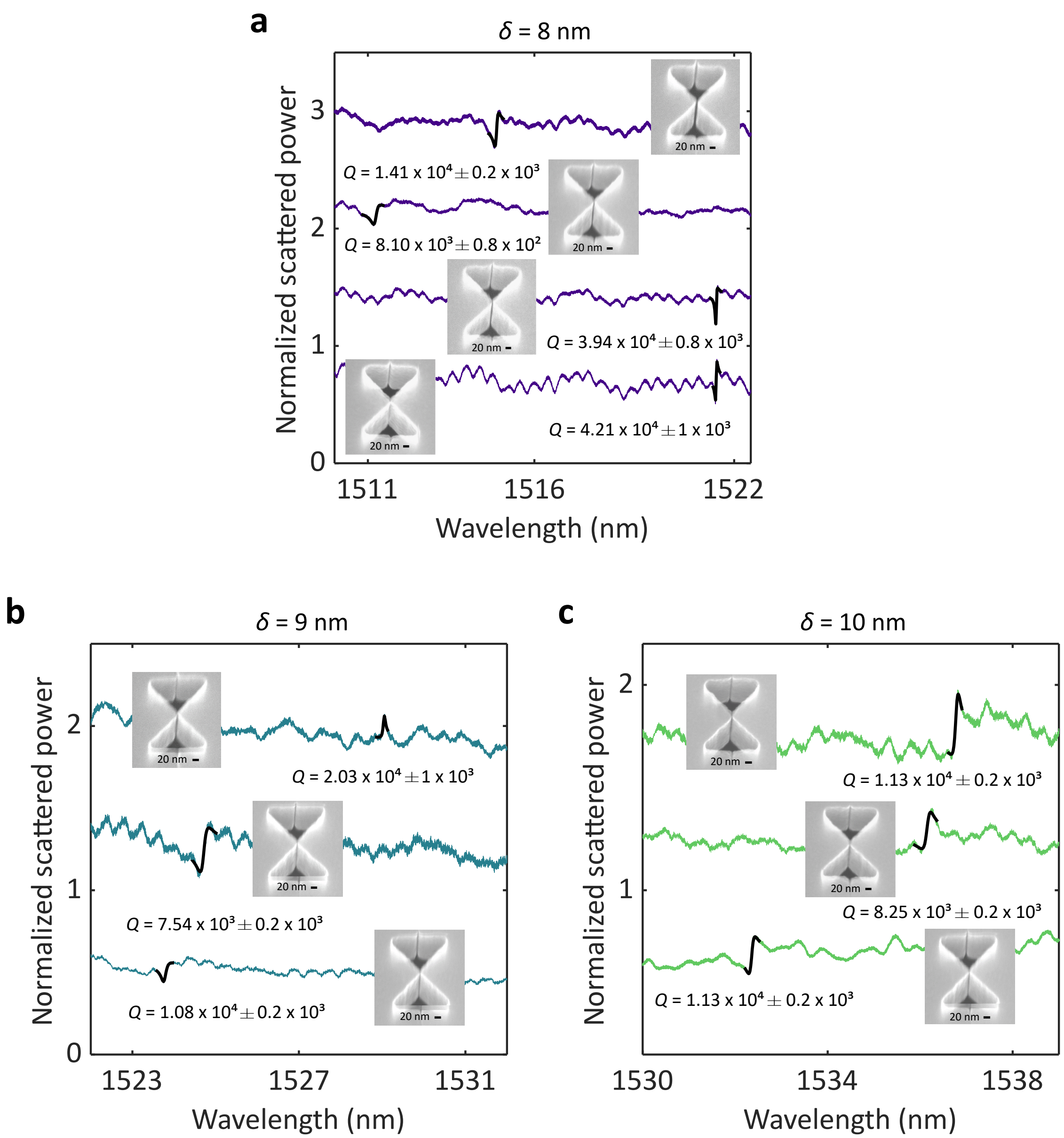

Supplementary Figure S29. **Resonant scattering from self-assembled nanobeam cavities. a-c**, Normalized scattering spectra of three sets of nominally identical cavities with offsets δ = 8, 9, and 10 nm, respectively. On each panel, the spectra are shifted vertically for clarity, and the resonant modes are fitted with Fano lineshapes (black lines). The extracted Q-factors along with tilted-view SEMs of the central bowtie unit cells are indicated in the plots.

modes of these five identical devices are plotted in Fig. S34, including the transmittance values, quality factors, and R^2 (goodness of fit). We observe a reduction of the quality factor and the on-resonance transmission, T_o, for all the cavities relative to the simulated values, respectively $Q = 4 \times 10^4$ and $T_o = 0.96$. We attribute the drop in Q to positional disorder

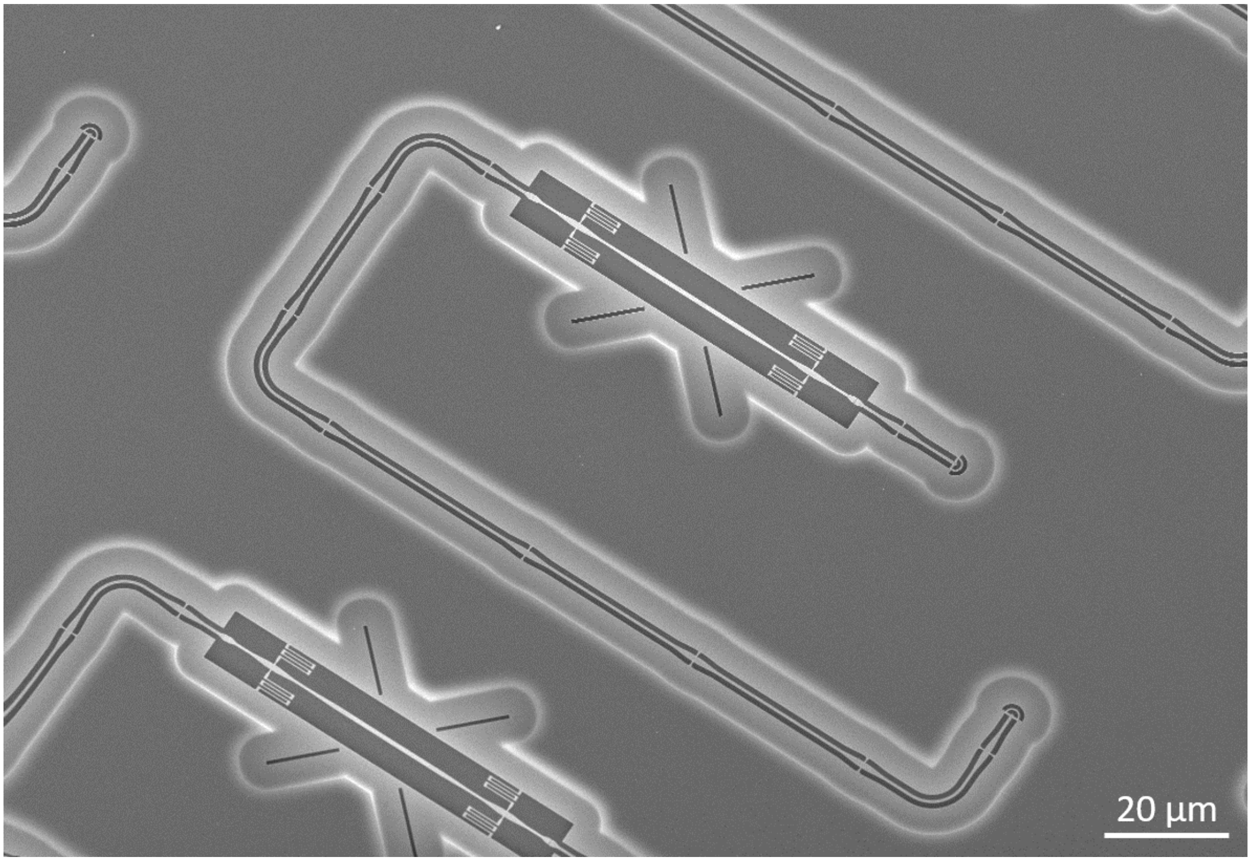

Supplementary Figure S30. **Suspended photonic circuits with self-assembled nanobeam cavities.** Tilted-view (20°) SEM image of a photonic circuit to characterize a self-assembled nanobeam cavity via in-plane transmission measurements.

and sidewall roughness, as for the cavities employed for resonant scattering measurements discussed in Section S5.1. The same mechanisms may explain the drop in T_o, although a non-negligible contribution may come from the differential bowing of the structure observed for the nanobeam cavities and the nanobeam waveguide, i.e., the normalization is not perfect. In addition, the measured Q and T_o fluctuate considerably, indicating a subtle interplay between the disorder-induced losses in the structure plane and the out-of-plane direction.

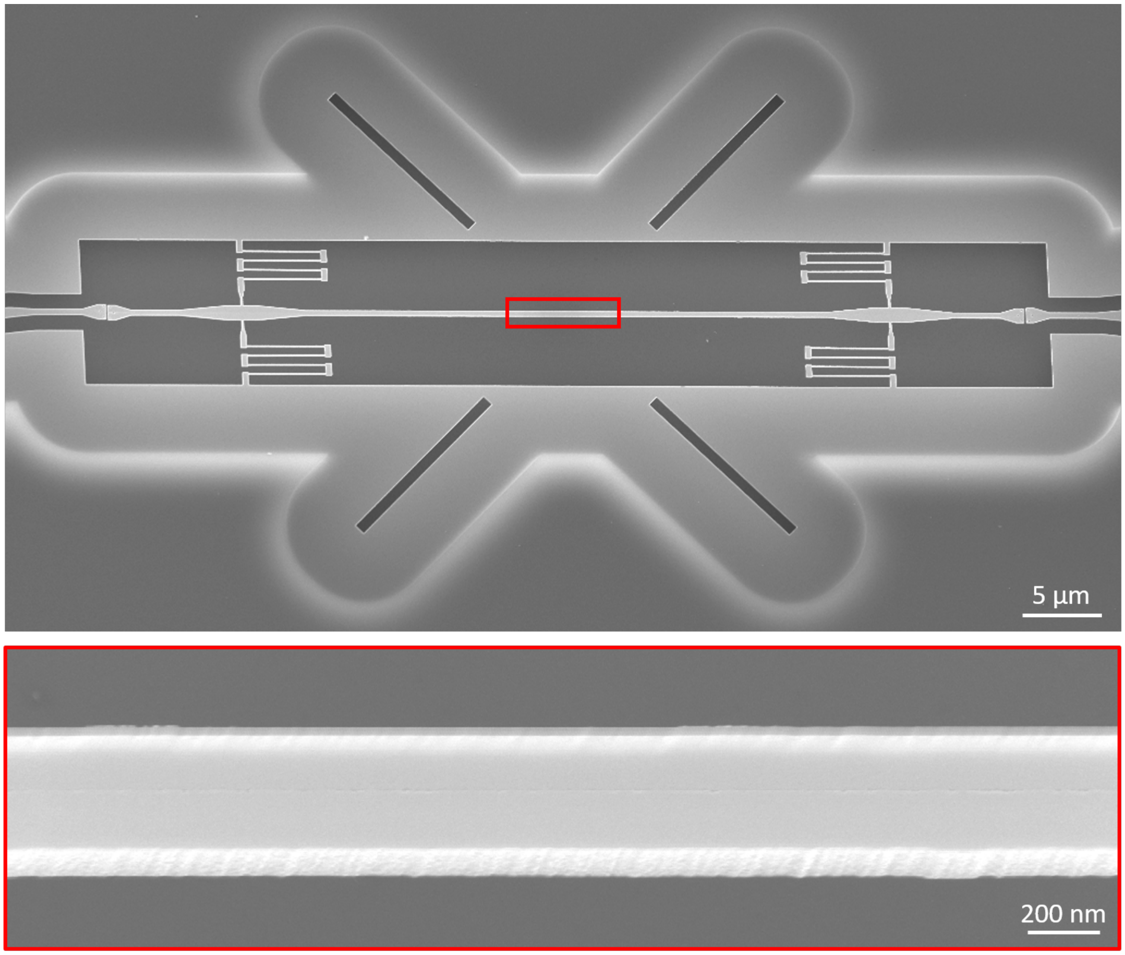

Supplementary Figure S31. **Suspended self-assembled nanobeam waveguides.** Tilted-view (20°) SEM image of a self-assembled nanobeam waveguide, the transmission of which is used to normalize the transmission measurements on self-assembled cavities. The red box is a zoom-in of the central part of the self-assembled waveguide.

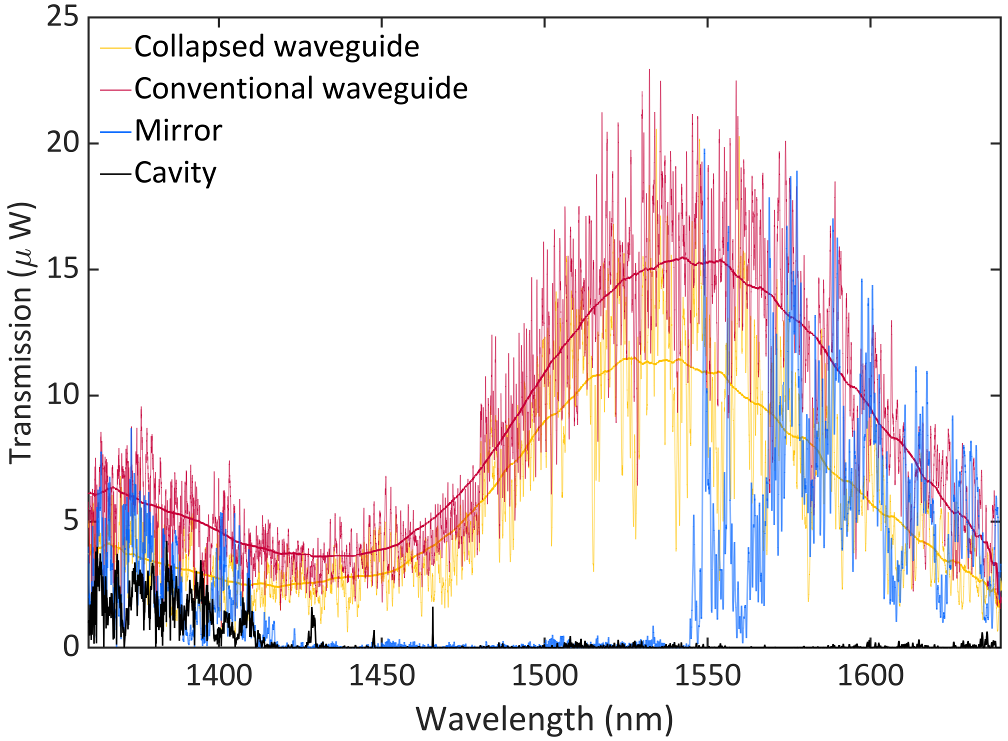

Supplementary Figure S32. **Raw and smoothed optical transmission spectra.** Transmission spectra of four devices with equivalent lengths: a self-assembled nanobeam waveguide, a self-assembled bowtie mirror, a self-assembled bowtie nanobeam resonator, and a conventional nanobeam waveguide. All spectra are taken at a laser power of $P = 10$ mW, and a smoothed curve is given for both the self-assembled and conventional nanobeam waveguides.

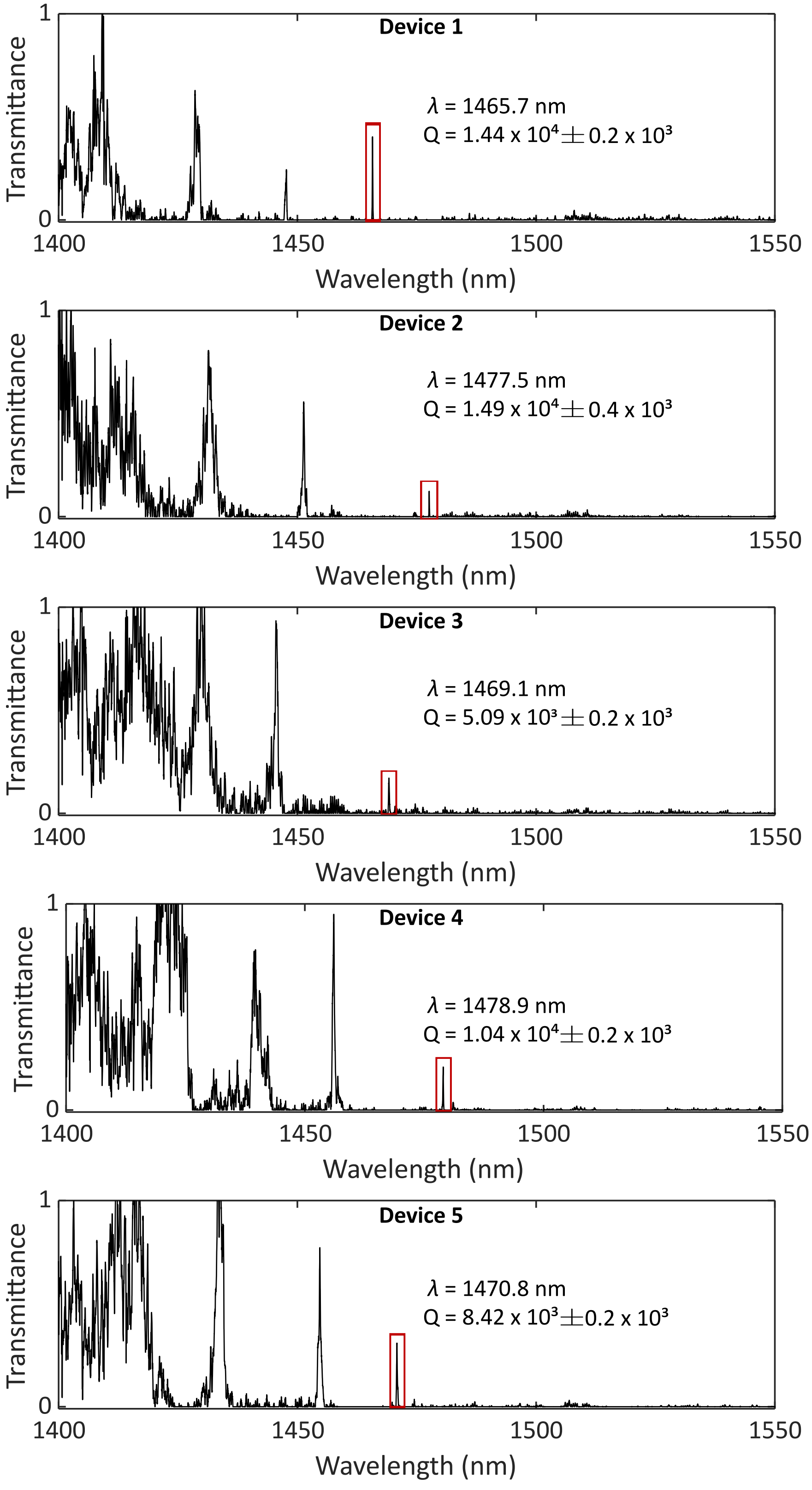

Supplementary Figure S33. **Optical spectroscopy of nominally identical waveguide-coupled self-assembled nanobeam cavities.** Transmittance spectra of 5 nominally identical bowtie cavities with an approximate bowtie width of 2 nm for $\delta = 12$ nm.

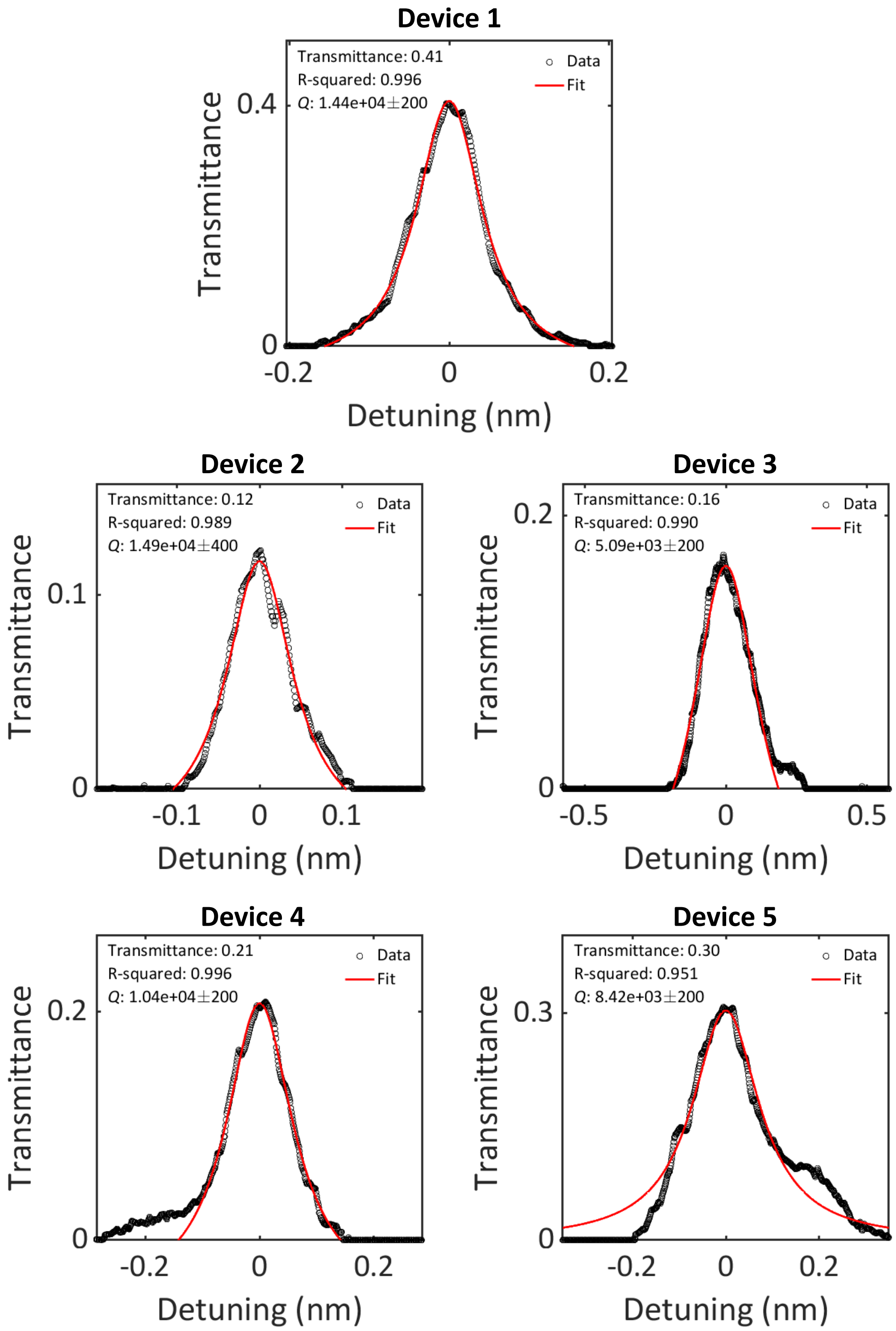

Supplementary Figure S34. **Lorentzian fit for self-assembled nanobeam cavities.** Fundamental cavity resonance and the associated Lorentzian fit. The extracted transmittance, R^2 (goodness of fit), and loaded quality factor are indicated.

* anaba@dtu.dk

† ssto@dtu.dk

[1] Liu, Y. et al. Promises and prospects of two-dimensional transistors. *Nature* **591**, 43–53 (2021).

[2] Aoyama, H. et al. The International Roadmap For Devices And Systems (IEEE, 2022); `https://irds.ieee.org/images/files/pdf/2022/2022IRDS_Litho.pdf`.

[3] Panuski, C. L. et al. A full degree-of-freedom spatiotemporal light modulator. *Nat. Photon.* **16**, 834–842 (2022).

[4] Minkov, M., Savona, V. & Gerace, D. Photonic crystal slab cavity simultaneously optimized for ultra-high Q/V and vertical radiation coupling. *Appl. Phys. Lett.* **111**, 131104 (2017).

[5] Gusso, A. & Delben, G. J. Dispersion force for materials relevant for micro-and nanodevices fabrication. *J. Phys. D* **41**, 175405 (2008).

[6] Hopcroft, M. A., Nix, W. D. & Kenny, T. W. What is the young's modulus of silicon? *J. Microelectromech. Syst.* **19**, 229–238 (2010).

[7] Casimir, H. B. On the attraction between two perfectly conducting plates. In *Proc. Kon. Ned. Akad. Wet.*, vol. 51, 793 (1948).

[8] Derjaguin, B., Abrikosova, I. & Lifshitz, E. Direct measurement of molecular attraction between solids separated by a narrow gap. *Q. Rev. Chem. Soc.* **10**, 295–329 (1956).

[9] Li, Y., Milton, K. A., Guo, X., Kennedy, G. & Fulling, S. A. Casimir forces in inhomogeneous media: Renormalization and the principle of virtual work. *Phys. Rev. D* **99**, 125004 (2019).

[10] Moazzami Gudarzi, M. & Aboutalebi, S. H. Self-consistent dielectric functions of materials: Toward accurate computation of Casimir–van der Waals forces. *Sci. Adv.* **7**, eabg2272 (2021).

[11] Quan, Q. & Loncar, M. Deterministic design of wavelength scale, ultra-high Q photonic crystal nanobeam cavities. *Opt. Express* **19**, 18529–18542 (2011).

[12] Kristensen, P. T., Van Vlack, C. & Hughes, S. Generalized effective mode volume for leaky optical cavities. *Opt. Lett.* **37**, 1649–1651 (2012).

[13] Albrechtsen, M. et al. Nanometer-scale photon confinement in topology-optimized dielectric cavities. *Nat. Commun.* **13**, 6281 (2022).

[14] Albrechtsen, M., Vosoughi Lahijani, B. & Stobbe, S. Two regimes of confinement in photonic

nanocavities: bulk confinement versus lightning rods. *Opt. Express* **30**, 15458–15469 (2022).

[15] Nasr Esfahani, M. et al. Effect of native oxide on stress in silicon nanowires: Implications for nanoelectromechanical systems. *ACS Appl. Nano Mater.* **5**, 13276–13285 (2022).

[16] Bohling, C. & Sigmund, W. Self-limitation of native oxides explained. *Silicon* **8**, 339–343 (2016).

[17] Rosiek, C. A. et al. Observation of strong backscattering in valley-hall photonic topological interface modes. *Nat. Photon.* 1–7 (2023).

[18] Arregui, G. et al. Cavity optomechanics with Anderson-localized optical modes. *Phys. Rev. Lett.* **130**, 043802 (2023).

[19] Loth, F., Kiel, T., Busch, K. & Kristensen, P. T. Surface roughness in finite-element meshes: application to plasmonic nanostructures. *JOSA B* **40**, B1–B7 (2023).

[20] Arregui, G., Gomis-Bresco, J., Sotomayor-Torres, C. M. & Garcia, P. D. Quantifying the Robustness of Topological Slow Light. *Phys. Rev. Lett.* **126**, 027403 (2021).

[21] Fukuda, A., Asano, T., Kawakatsu, T., Takahashi, Y. & Noda, S. Suppressing the sample-to-sample variation of photonic crystal nanocavity Q-factors by air-hole patterns with broken mirror symmetry. *Opt. Express* **31**, 15495–15513 (2023).

[22] Minkov, M., Dharanipathy, U. P., Houdré, R. & Savona, V. Statistics of the disorder-induced losses of high-Q photonic crystal cavities. *Opt. Express* **21**, 28233–28245 (2013).

[23] GarcÃa, P. D., Javadi, A., Thyrrestrup, H. & Lodahl, P. Quantifying the intrinsic amount of fabrication disorder in photonic-crystal waveguides from optical far-field intensity measurements. *Applied Physics Letters* **102**, 031101 (2013).

[24] Nguyen, V. T. H. et al. The core sequence: A nanoscale fluorocarbon-free silicon plasma etch process based on SF6/O2 cycles with excellent 3D profile control at room temperature. *ECS J. Solid State Sci. Technol.* **9**, 024002 (2020).

[25] Cuffe, J. et al. Fine control of critical dimension for the fabrication of large bandgap high frequency photonic and phononic crystals. *Microelectron. Eng.* **88**, 2233–2235 (2011).

[26] Nguyen, V. T. H. et al. Ultrahigh aspect ratio etching of silicon in SF6-O2 plasma: The clear-oxidize-remove-etch (CORE) sequence and chromium mask. *J. Vac. Sci. Technol. A* **38**, 053002 (2020).

[27] Arregui, G. et al. Cavity Optomechanics with Anderson-Localized Optical Modes. *Phys. Rev. Lett.* **130**, 043802 (2023).

[28] Hoang Nguyen, V. T. et al. Cr and CrOx etching using SF6 and O2 plasma. *J. Vac. Sci. Technol. B* **39**, 032201 (2021).

[29] Galli, M. et al. Light scattering and Fano resonances in high-Q photonic crystal nanocavities. *Appl. Phys. Lett.* **94**, 071101 (2009).
